# Topological cluster statistic (TCS): Toward structural connectivity–guided fMRI cluster enhancement

**DOI:** 10.1162/netn_a_00375

**Published:** 2024-10-01

**Authors:** Sina Mansour L., Caio Seguin, Anderson M. Winkler, Stephanie Noble, Andrew Zalesky

**Affiliations:** Department of Biomedical Engineering, The University of Melbourne, Melbourne, Victoria, Australia; Melbourne Neuropsychiatry Centre, The University of Melbourne, Melbourne, Victoria, Australia; Department of Psychological and Brain Sciences, Indiana University, Bloomington, IN, United States; National Institute of Mental Health, National Institutes of Health, Bethesda, MD, United States; Department of Psychology, Department of Bioengineering, Center for Cognitive and Brain Health, Northeastern University, Boston MA, United States

**Keywords:** fMRI, Brain activation, Structural connectivity, Cluster inference, Power, Human connectome

## Abstract

Functional magnetic resonance imaging (fMRI) studies most commonly use cluster-based inference to detect local changes in brain activity. Insufficient statistical power and disproportionate false-positive rates reportedly hinder optimal inference. We propose a structural connectivity–guided clustering framework, called topological cluster statistic (TCS), that enhances sensitivity by leveraging white matter anatomical connectivity information. TCS harnesses multimodal information from diffusion tractography and functional imaging to improve task fMRI activation inference. Compared to conventional approaches, TCS consistently improves power over a wide range of effects. This improvement results in a 10%–50% increase in local sensitivity with the greatest gains for medium-sized effects. TCS additionally enables inspection of underlying anatomical networks and thus uncovers knowledge regarding the anatomical underpinnings of brain activation. This novel approach is made available in the PALM software to facilitate usability. Given the increasing recognition that activation reflects widespread, coordinated processes, TCS provides a way to integrate the known structure underlying widespread activations into neuroimaging analyses moving forward.

## Author Summary

Neuroimaging studies often encounter challenges in reliable inference of statistical maps due to limited statistical power. This article introduces TCS, a novel method that integrates anatomical connectivity data from diffusion tractography into cluster-based inference techniques. Our findings demonstrate that TCS enhances statistical power, improves the detection of spatially disjoint localized activations, and identifies the underlying network linking distant inferred active regions. By elucidating the coordinated network supporting inferred effects, TCS enables data-driven interpretation of inference results. The availability of TCS as a publicly accessible tool offers a promising avenue for future neuroimaging research to leverage anatomical connectivity for enhanced inference and interpretation.

## INTRODUCTION

Functional magnetic resonance imaging (fMRI) has emerged as a prominent noninvasive tool to study the functional organization of the human brain (Glover, 2011; Heeger & Ress, 2002; Logothetis, 2008). Using fMRI we can obtain high-resolution statistical maps indicating differences in localized brain activity. For instance, a statistical map can be computed to quantify the difference in brain activity while performing a certain task compared to rest. Brain-wide statistical inference of activation from such maps remains a fundamental goal for fMRI studies. The high dimensionality and limited sample sizes of neuroimaging datasets hinder the ability to perform accurate inferences (Cremers, Wager, & Yarkoni, 2017; Eklund, Nichols, & Knutsson, 2016). Due to the large multiplicity of tests (e.g., voxels), direct inference from uncorrected statistical maps yields increased false-positive rates (type I error) (Carp, 2012). On the other hand, dependencies across multiple tests render traditional univariate correction approaches (such as the Bonferroni method) too stringent, and thus underpowered (higher type II error).

One of the most common methods to enhance statistical power while controlling error rates is to group spatially contiguous regions showing a suprathreshold effect into clusters and define a cluster-level statistic (Bullmore et al., 1999; Forman et al., 1995; Friston, Worsley, Frackowiak, Mazziotta, & Evans, 1994). The null hypothesis can then be tested for each cluster using random field theory (Worsley, Evans, Marrett, & Neelin, 1992; Worsley, Marrett, Neelin, & Evans, 1996; Worsley, Marrett, Neelin, Vandal, et al., 1996) or permutation testing (Nichols & Holmes, 2002; Winkler, Ridgway, Webster, Smith, & Nichols, 2014), while controlling the family-wise error rate (FWER) or false discovery rate (FDR). Inference at the spatial scale of clusters is meaningful because observing an effect in multiple spatially proximal voxels increases our confidence that the effect is not spurious, since true task-related signal is more likely to be spatially extended than random noise.

Altogether, cluster-based inference improves power compared to traditional univariate inference by leveraging the fact that effects tend to occur in spatially contiguous clusters of voxels. However, it is well-established that functional systems, such as the default mode and fronto-parietal networks, are also characterized by spatially distributed patterns of activation, in which distant, noncontiguous groups of voxels show correlated activity (Yeo et al., 2011). The concerted activity of spatially distributed functional systems is facilitated by the brain’s underlying white matter connectivity (Hagmann et al., 2008; Honey et al., 2009; Sarwar, Tian, Yeo, Ramamohanarao, & Zalesky, 2021; van den Heuvel, Mandl, Kahn, & Hulshoof Pol, 2009). Anatomical connections support communication between distant brain areas, thus enabling the coordinated activity of noncontiguous groups of gray matter voxels (Liu et al., 2022; Seguin, Mansour L., Sporn, Zalesky, & Calamante, 2022). While some have explored the impact of grouping noncontiguous areas, there have not yet been any attempts to group areas based on the known underlying anatomy (Noble, Mejia, Zalesky, & Scheinost, 2022).

Here, we propose the topological cluster statistic (TCS), a novel cluster-based inference method that takes into account both the spatial proximity and the underlying structural connectivity between voxels. Leveraging advances in high-resolution connectomics (Mansour L., Seguin, Smith, & Zalesky, 2022; Mansour L., Tian, Yeo, Cropley, & Zalesky, 2021), distinct voxels showing a significant effect are grouped in the same cluster if they are spatially proximal or anatomically connected, as inferred using tractography and diffusion MRI. As such, TCS allows for the identification of spatially distributed yet anatomically interconnected clusters.

We evaluate the performance of TCS using both simulated and real fMRI data, comparing the sensitivity and specificity of TCS to established—white matter agnostic—cluster-based methods. We test the hypothesis that exploiting structural connectivity increases statistical power, thus enabling the detection of a greater number of true positive effects at equal sample sizes, in comparison to current methods. In addition, we explore how our approach can enhance the interpretation of statistical maps by grouping spatially disjoint clusters through underlying anatomical networks.

## RESULTS

**Figure 1.**
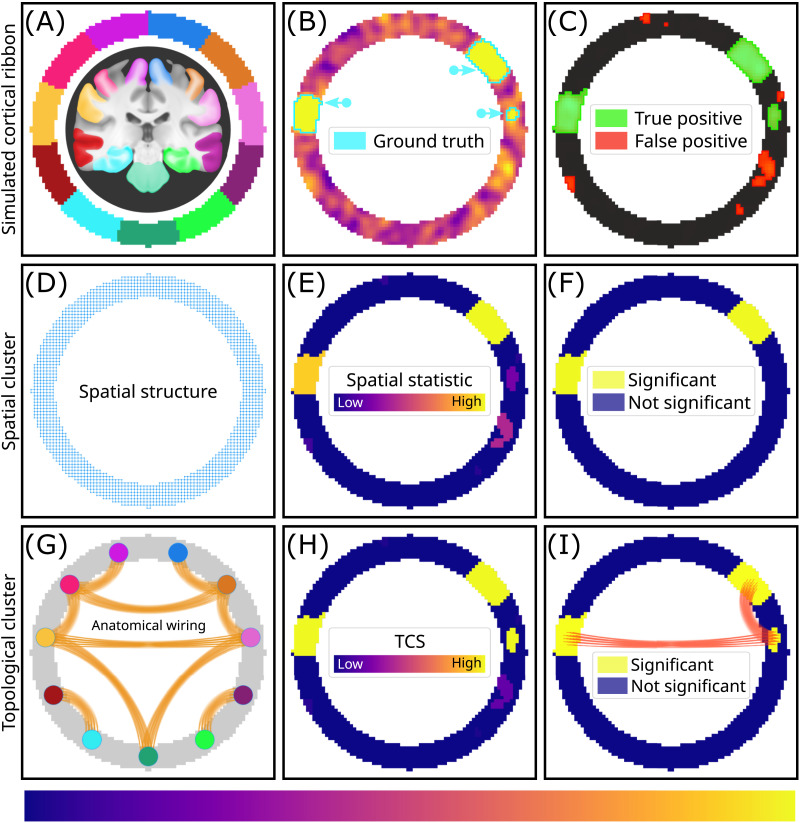
A hypothetical simulation illustrating TCS advantages. (A) A representative axial slice from the brain presented as a circular ribbon indicating different gray matter regions from a brain atlas. (B) An example statistical map. (C) The thresholded map indicating suprathreshold effects, color-coded to true positives (green) and false positives (red). (D) Spatial structure used in traditional cluster inferences. (E) Spatial cluster-size statistic groups spatially contiguous voxels into clusters. The number of voxels in a cluster can be used to determine the cluster-level statistical significance. (F) Regions that survive statistical correction of the spatial statistic. (G) Anatomical connectivity phantom of white matter connections. (H) Topological cluster-size statistic (TCS) enables grouping spatially disjoint clusters based on anatomical priors. The three disjoint spatial clusters receive the same cluster label due to anatomical linkage. (I) Regions that survive correction with TCS and the underlying anatomical wiring.

We first demonstrate the motivation behind TCS and its potential advantages on an example simulation. Figure 1 provides an illustrative example comparing the operation of TCS to established cluster-based methods. A two-dimensional image is used to represent an axial slice of the human brain and a ribbon is used to represent gray-matter voxels parcellated into different brain regions (Figure 1A). A signal is simulated for each pixel within the ribbon. Specifically, three regions of significant effect varying in spatial extent (marked by a cyan outline) are contaminated with noise and spatial autocorrelation to represent a sample statistical map (Figure 1B). A cluster-defining threshold (CDT) is used to locate candidate significant voxels. While active regions from the ground truth (green mask in Figure 1C) survive after thresholding, several spurious regions remain (red mask in Figure 1C).

Cluster-based approaches group suprathreshold voxels according to a spatial adjacency structure (Figure 1D). First, the size of each cluster is determined based on the number of suprathreshold voxels it comprises (a cluster statistic, Figure 1E). This statistic is then assessed for significance in relation to a null model (Figure 1F). The null indicates the likelihood of finding a cluster of a particular size in the absence of any true signal (clusters formed by spatially autocorrelated noise artefacts). We see that smaller active regions are not inferred as significant with spatial clustering approaches.

TCS requires an anatomical connectivity map. In this example, we constructed an illustrative network resembling anatomical connections from association fibers (short-range connections within a lobe), commissural fibers (homotopic connections between the left and right hemispheres), and projection fibers (connecting the brain stem to the cortex) (Figure 1G). Unlike cluster-based approaches, TCS identifies clusters based on an adjacency structure that takes into account both binary anatomical connectivity and spatial proximity. Suprathreshold voxels are clustered based on spatial proximity and anatomical connectivity to yield clusters that can be spatially disjoint. The total number of voxels comprising each cluster defines the cluster’s test statistic (Figure 1H). This example illustrates how TCS detects the small cluster that is not detected by cluster-based methods and additionally provides insights into underlying anatomical connectivity (Figure 1I). Next, we conducted an empirical investigation of the potential benefits of TCS on task fMRI activation statistical maps.

### Task fMRI Data

**Figure 2.**
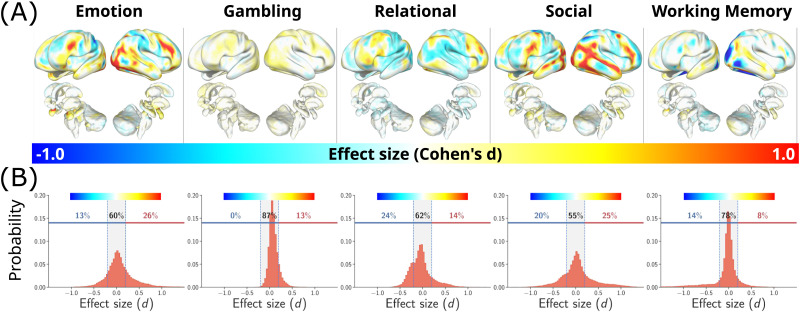
Effect sizes and spatial distributions of brain activation maps for five tasks. (A) Spatial distribution of statistical maps of activation for different tasks and contrasts. Intensities indicate the effect size of observed effects at each voxel/vertex. Positive effects (warm colors) indicate activation and negative effects (cold colors) mark the deactivation of regions relative to task contrast. (B) Histograms illustrate the probability distribution of effects with varying sizes. The gray-shaded region indicates small effects (∣*d*∣ < 0.2). The percentage of voxels with small effect sizes (∣*d*∣ < 0.2), and positive and negative effects exceeding this threshold are reported.

Task fMRI data was sourced from the Human Connectome Project (HCP) (Van Essen et al., 2013). Five tasks were selected to enable the investigation of various brain activation patterns and effect sizes (see Methods for detail). A putative ground truth effect size at each brainordinate (voxel/vertex) was estimated from a group-level contrast for the full sample (*N* = 983–994), as described previously (Cremers et al., 2017; Noble, Scheinost, & Constable, 2020). A one-sample *t* statistic was then computed for each brainordinate and transformed to Cohen’s *d* to estimate an effect size (Figure 2A). Effect sizes were small (∣*d*∣ < 0.2) for the majority of brainordinates (55% to 87%, Figure 2B).

Whole-brain anatomical connectivity for TCS was inferred with probabilistic tractography of diffusion-weighted imaging data. High-resolution structural connectivity (Mansour L., Tian, et al., 2021) was mapped for all HCP individuals (*N* = 1,000, 91,282 brainordinates). A smooth distance-dependent group consensus structural connectome was then constructed to represent normative anatomical connectivity structure while preserving long-range connections (Betzel, Griffa, Hagmann, & Mišić, 2019; Mansour L. et al., 2022; Roberts, Perry, Roberts, Mitchell, & Breakspear, 2017). This structure was combined with existing spatial neighborhood information to form the topology used in TCS to cluster active brain regions (see Methods for detail). It is noteworthy to highlight that other alternative connectivity maps can be utilized with TCS, including individual connectomes, which could capture detailed personalized effects. Here, we used a group-level connectome to derive a fair comparison with conventional group-level cluster inferences.

### Evaluating Statistical Power

Inference cannot be made about individual voxels comprising a cluster. We can only claim that there exists at least one voxel within a cluster for which the null hypothesis can be rejected. Nevertheless, it is possible to evaluate the statistical power of clustering methods at the granularity of voxels, since accurate detection of a cluster necessitates accurate delineation of its constituent voxels. Hence, we used a notion of power/sensitivity that measures the likelihood of detecting true effects (Noble et al., 2020) in suprathreshold clusters. To evaluate statistical power, we randomly sampled 40 individuals from the full cohort to represent a nominal study sample. TCS and spatial cluster–based statistics were then used to identify significant clusters of changes in brain activation for each of the five tasks, controlling the FWER at 5% for each task. This was repeated for 500 random samples of 40 individuals. To quantify statistical power, the set of brainordinates comprising each cluster was compared to the putative ground truth defined using the full cohort. Specifically, for each brainordinate, statistical power was computed based on the proportion of random samples in which (a) the group-level effect sign (positive/negative) was in agreement with the ground truth effect, and (b) the brainordinate belonged to a cluster that survived multiple comparison correction at the level of cluster statistic.

**Figure 3.**
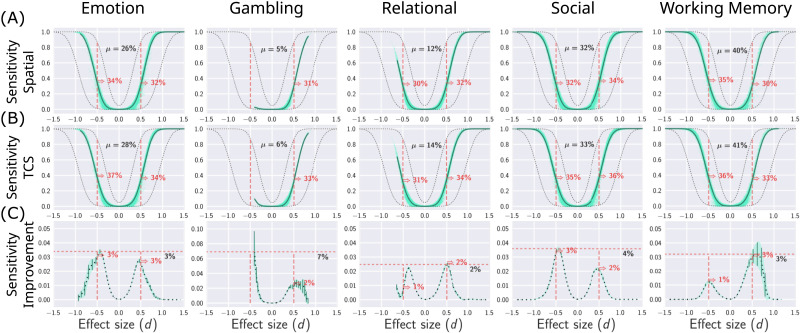
Evaluation of mean sensitivity of TCS and (spatial) cluster-based statistics as a function of the ground truth effect size. The vertical axes show statistical power (true positive rates) and the horizontal axes show effect sizes defined by the putative ground truth. Each column denotes a distinct task. The top and middle rows (A, B) correspond to a cluster-based statistic and TCS, respectively. The statistical power at every brainordinate is depicted by a single scatter point. The bottom row (C) quantified the absolute improvement in statistical power achieved with TCS (the difference in means of the first two rows). The mean sensitivity against effect size was computed by fitting cubic splines to a moving average. The dashed gray lines (A, B) indicate estimates from power analyses for a one-sample *t* test without any correction (*p* < 0.05, upper line) and after Bonferroni correction (*p* < $\frac{0.05}{N_{b}}$, lower line) in a sample of *N* = 40 (*N*_*b*_ = 91,282 denotes the number of brainordinates). *μ* indicates the mean sensitivity for suprathreshold effects (∣*d*∣ > 0.2). In the bottom row, the maximum mean improvements are depicted by horizontal dashed lines, the improvement at medium effects (∣*d*∣ = 0.5) are additionally presented, and the 95% confidence interval is marked by the shaded green curve.

Figure 3 shows statistical power as a function of the putative ground truth effect size at each brainordinate. As expected, for both TCS and the cluster-based method, the statistical power increases with effect-size magnitude. For medium-sized effects (∣*d*∣ = 0.5), on average, TCS yielded a 3%–5% increase in sensitivity than the cluster-based statistic (depicted by the dashed vertical lines in Figure 3). The extent of average improvement varies across tasks and effect sizes. The third row shows the sensitivity improvement across tasks and effect sizes. This indicates the degree of improvement attained by TCS at different effect sizes. Most importantly, we observed a significant yet modest increase in the statistical power of detecting various effects ranging from small to large sizes (0.2 < ∣*d*∣ < 0.8) with a maximum average improvement of 4%–10% observed for medium-sized effects. These results suggest that considering anatomical connectivity in forming cluster-based statistics consistently increases the average (brain-wide) statistical power of these inferences. Supplemental analyses demonstrated that these findings were consistent on varying study sample sizes and cluster-defining thresholds (see Supporting Information Figures S1 and S2). In the case of the gambling task, our supplemental analyses resulted in wider confidence intervals that may be due to less spatially distributed strong effects for this particular task, as well as greater variability across participants in task performance, compared to the other tasks.

### Localized Gains in Sensitivity

**Figure 4.**
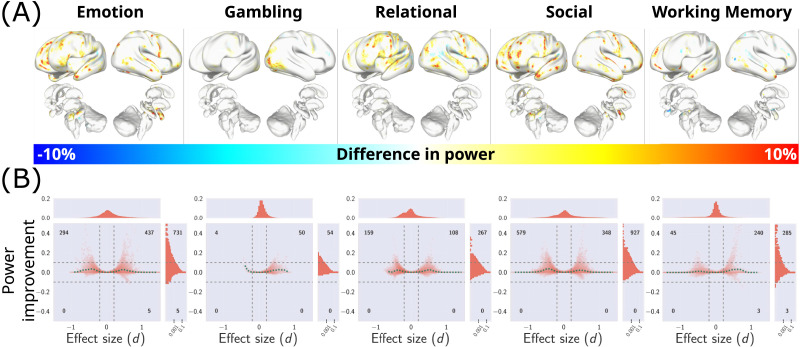
Evaluation of localized sensitivity improvements achieved by TCS. (A) The vertex-wise gains in sensitivity improvement of TCS compared to cluster-based statistics are projected onto a surface representation of the brain. Warm colors indicate brainordinates where using TCS resulted in a sensitivity improvement. Conversely, cool colors indicate regions where cluster-based statistics provided higher sensitivity. The color maps are capped at 10% change in sensitivity. (B) A 2D histogram (heatmap) indicating the distribution of vertex-wise sensitivity improvements against ground truth effect size. The marginal distributions are depicted on the sides of the heatmap. Dashed vertical lines depict the small effect threshold (∣*d*∣ = 0.2) and dashed horizontal lines depict a 10% change in sensitivity. The number of brainordinates exceeding both vertical and horizontal thresholds are presented on the four corners of the heat map. The dashed curve depicts the mean improvements (similar to Figure 3C). The marginal distribution of sensitivity improvement (histograms on the right side of the heat map) is presented on a logarithmic scale.

Results presented in Figure 3 illustrated the consistent improvement in average sensitivity (as a function of effect size) achieved after TCS. We next sought to assess the distribution of localized brain-wide power improvements. To this end, sensitivity improvements (i.e., Figure 3C) at the resolution of brainordinates were projected on a surface representation of the brain (depicted in Figure 4A). This revealed that TCS resulted in widespread sensitivity improvements of more than 10% for a range of cortical and subcortical brain regions. A 2D histogram of sensitivity changes after TCS (Figure 4B) indicated up to 40% power improvements in certain brainordinates. These results also demonstrated a relative loss of more than 10% sensitivity in certain loci. Nevertheless, the proportion of brainordinates suffering from this loss in sensitivity is consistently smaller than those benefiting from sensitivity gains achieved by TCS. These results suggest that TCS can selectively improve the statistical power of clustering approaches in finding localized differences that are better supported by the underlying anatomical connectivity. Supplementary analyses of the local gains in sensitivity replicated these results on a range of alternative sample sizes and cluster-defining thresholds (see Supporting Information Figures S3, S4, S5, and S6).

### Evaluating Sensitivity-Specificity Balance

**Figure 5.**
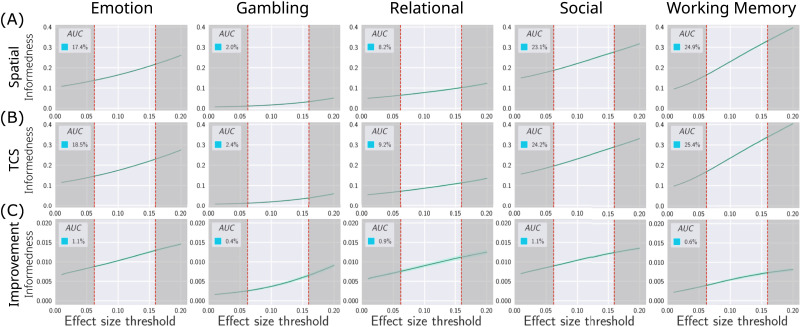
Informedness of clustering inference approaches. Significant clusters surviving correction were treated as labels for binary classification (significant vs. null). A threshold is used to yield binary labels from the putative ground truth. We assume that all suprathreshold effects should be classified as significant. The bookmaker informedness index was utilized to compare performance. The top row (A) shows the informedness of spatial clustering and TCS as a function of the binarization threshold. Informedness was summarized by the normalized area under the curve (AUC) that averages informedness across thresholds, thus providing a threshold-independent comparison. The bottom row (B) indicates the informedness improvement yielded by TCS. The shaded lines, albeit occasionally narrow, represent the 95% confidence interval. The dashed vertical lines indicate the required effect for statistical significance (*α* = 5%) in the putative ground truth sample (*N* = 1,000) with (right) and without (left) a Bonferroni correction. The right line indicates a very stringent binarization threshold, and the left line gives a more lenient threshold. AUC was computed for values falling between the two dashed lines (see Methods for further detail).

It is important to ensure that any gain in sensitivity is not at the expense of a greater loss in specificity. In other words, an inference method should (i) detect brain regions that show significant changes in activation, and (ii) discard regions that do not express considerable changes in activation. To assess the sensitivity improvements of the inference classifier while accounting for changes in specificity, the bookmaker informedness index was utilized (Youden, 1950). This measure indicates the accuracy of a classifier compared to chance while accounting for specificity (Powers, 2020), thus providing an unbiased classification metric (Luque, Carrasco, Martín, & de las Heras, 2019; Zhu, 2020) (see Methods for detail). Succinctly, this measure falls in the range of −1 (completely incorrect classification) to 1 (perfect classification) and is linearly proportional to balanced accuracy (*BI* = *TPR* + *TNR* − 1; see section on Classifier Informedness). Figure 5 provides a comparison of informedness curves for spatial cluster-based statistics and TCS approaches for different binarization thresholds.

Figure 5A and B shows the respective informedness of spatial and TCS inferences. For all tested thresholds, the difference in informedness is reported in the third row. This comparison shows that TCS yields significant increases in informedness across a range of valid effect thresholds. The gain in sensitivity achieved by TCS was more than any potential losses in specificity and this resulted in a better informed classifier. Additionally, we observe a uniformly increasing trend in the classification performance of all models with an increase in the binarization threshold. This shows that the classification of effects with higher magnitudes is more informed regardless of the clustering statistic. Supplemental analyses show that these findings were consistently replicable on varying study sample sizes, cluster-defining thresholds, and alternative evaluations of inference performance (see Supporting Information Figures S7, S8, S9, and S10).

### Case Study: TCS Advantages in a Single Task

**Figure 6.**
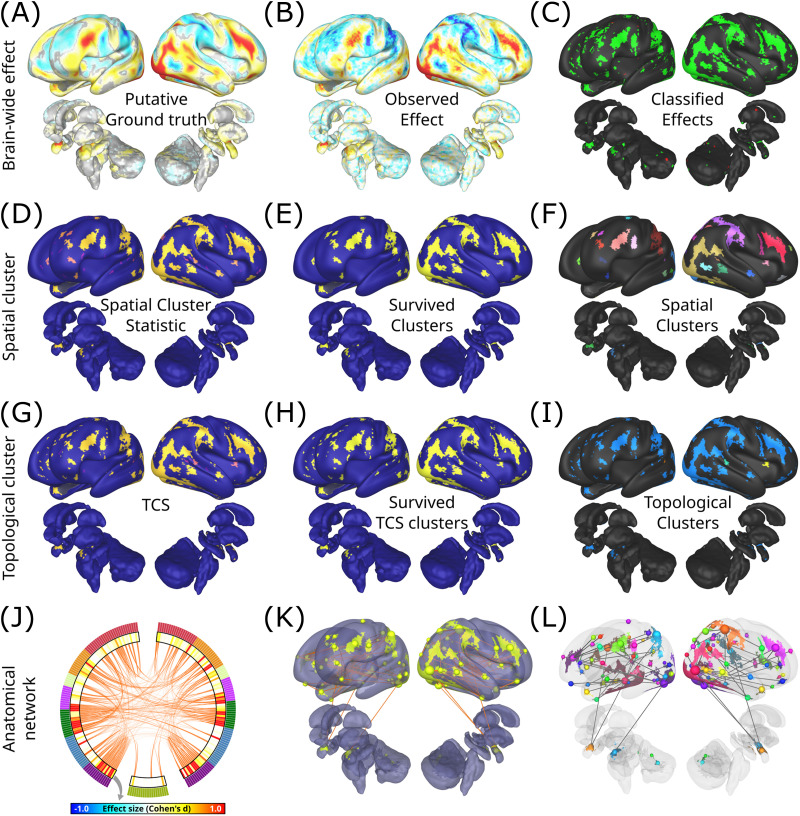
Illustrating benefits of TCS on a single task. (A) The putative ground truth effect size (*N* = 988) for the emotion task. The effects not reaching significance (*p* ≥ 0.05) are grayed out. (B) The effects from the same task, observed over a smaller sample (*N* = 40). (C) The effects are colored to distinguish between detectable true positives (green), false positives (red), and regions where the observed effect falls below the cluster-forming threshold (black). (D) The spatial cluster-based statistic value for all suprathreshold spatial clusters. (E) The spatial clusters that survive nonparametric FWER correction at the level of clusters. (F) Clusters colored by spatial contiguity. (G) TCS for suprathreshold regions. (H) TCS clusters that survive correction. (I) Clusters colored by topological contiguity. (J) A chord diagram of anatomical connectivity between TCS clusters downsampled to a brain atlas. The diagram is segmented into the subcortex and cerebellum (bottom), and the left and right cortices (colored by their respective seven RSN networks in the outer layer). The inner layer depicts the mean effect size. (K) Anatomical connectivity between spatially disjoint effect clusters. (L) The spanning tree covering the key anatomical connections based on maximal pairwise effect magnitude.

To present the key improvements of TCS over spatial cluster-based methods, we next focused on the emotion task (supplementary results are provided for the other four tasks; see Supporting Information Figures S11, S12, S13, S14, S15, and S16). We aimed to assess whether TCS can achieve its expected benefits (presented in Figure 1) on real-world fMRI data. Figure 6A, B, and C show that for a single repetition sample (*N* = 40) the observed suprathreshold effects can be compared with the putative ground truth to mark the desired classification outcome. With cluster-based statistics, only larger spatially contiguous clusters survive correction (Figure 6D and E); furthermore, the conventional cluster-based approach only provides a set of fragmented significant effects, which can be difficult to interpret (Figure 6F). In contrast, by leveraging anatomical information, TCS can detect true positive effects of a smaller contiguous spatial extent (Figure 6G and H, also see Supporting Information Figure S15); moreover, TCS reveals how most of these spatially disjoint effects belong to a unified anatomically linked cluster (Figure 6I).

This anatomically linked network can be further inspected to understand the underlying anatomical nature of the observed activations and reveal potential biological pathways involved in a particular task activation. In Figure 6J, the high-resolution structural connectivity between suprathreshold effects is downsampled to a well-established brain atlas comprising 200 cortical (Schaefer et al., 2018) and 19 subcortical and cerebellar structures (see Methods for detail). This presents a brain-wide interconnected network supporting the detected activity patterns. This network can be further probed to investigate the connectivity between major spatially contiguous clusters (Figure 6K, see Methods for detail). This network can additionally be filtered based on observed effect magnitudes to present a spanning tree of the most important underlying connections (Figure 6L). For the emotion task, consistent with previous literature, this filtered network depicts the importance of cortico-subcortical pathways of the amygdala in the perception of faces (Hadj-Bouziane et al., 2012; Leonard, Rolls, Wilson, & Baylis, 1985; McDonald, 1998; McFadyen, Mermillod, Mattingley, Halász, & Garrido, 2017; Pessoa & Adolphs, 2010). Thus, the amygdala acts as a bridging node between cortical activation clusters. Additionally, the network analysis highlights the central role of visual cortices (homotopic clusters containing the occipital face area, the fusiform face area, and the superior temporal sulcus) as the principal network hubs interconnecting the brain-wide pattern of activity as supported by distributed neural models of face perception (Haxby, Hoffman, & Gobbini, 2000; Tsao & Livingstone, 2008). These results demonstrate the interpretive gain of TCS in transforming fragmented areally distributed effects into structured anatomically informed activity networks.

## DISCUSSION

Brain activity is fundamentally shaped by the anatomical backbone supporting neural communication (Fornito, Zalesky, & Breakspear, 2013). The functional organization of the human brain is known to be closely related to its structural connectivity (Baum et al., 2020). Here, we introduced a new approach to detect spatially distributed signals that are interconnected by this underlying anatomical topology. The proposed method enhances the statistical power of widely used clustering approaches, especially in detecting effects of smaller spatial extent. TCS additionally enables inspection of the anatomical network implicated in an activation map.

### Increased Sensitivity

In our evaluations, we found that TCS improves the statistical power of detecting a wide range of effect sizes (0.2 < ∣*d*∣ < 0.8). In particular regions, more than 10% local improvements in power were observed. Importantly, the gain in sensitivity was achieved without detrimental effects on specificity and TCS provided a more informed inference model.

### Interpreting the Significance Network

In addition to the improvements in detecting localized activation patterns, TCS provides additional information by linking spatially disjoint effects into a unified anatomical network. This is a crucial feature that addresses potential limitations of existing localized inference approaches (Noble et al., 2022). As TCS uses a high-resolution anatomical topology, it can provide rich anatomical information about the underlying white matter fibers connecting an activation map. We have provided visualizations in the form of atlas brain networks and spatial cluster connectograms that yielded insights regarding the underlying anatomical connectivity. Such techniques facilitate anatomically guided interpretations of associations between brain activation and connectivity. Considering the importance of probing these structure-function relations in task fMRI studies, TCS strengthens the biological interpretations made from task fMRI inferences.

### Relationship With Sample Size and Cluster-Defining Threshold

Previous studies have emphasized the importance of adequate sample size to reliably detect effects of certain magnitudes. Similarly, our supplemental analyses highlight the importance of appropriate power analyses to determine sample size (Mumford & Nichols, 2008; Poldrack et al., 2017; Sullivan & Feinn, 2012; Turner, Paul, Miller, & Barbey, 2018). Our supplementary analyses also show the importance of the cluster-defining threshold (CDT) parameter that can impact the sensitivity of cluster-based inference approaches. Nevertheless, at any given sample size or CDT, TCS was able to improve the detection of effects in smaller spatially distributed regions that were anatomically connected to other suprathreshold regions. These smaller regions are commonly neglected with a spatial cluster–based inference method. Hence, TCS can improve the reported shortcomings of traditional cluster-based approaches in detecting small interconnected activations (Lohmann et al., 2018).

### TCS Generalization: Correction Methods

While our evaluation focused on comparing the proposed TCS enhancement with the widely used cluster-extent method (Friston et al., 1994), there exists an opportunity for TCS to expand its applicability to more advanced spatial inference methodologies, such as threshold-free cluster enhancement (TFCE) (Smith & Nichols, 2009). The documented advantages of TFCE-based methods (Noble et al., 2020), the growing adoption of these techniques (Acar et al., 2023), and their capacity to provide voxel-wise inferences collectively advocate for future investigations into the potential synergies between TCS and TFCE. We have incorporated TCS into the Permutation Analysis of Linear Models (PALM) tool, available from the FSL website at https://fsl.fmrib.ox.ac.uk/fsl/fslwiki/PALM (Winkler, Ridgway, Douaud, Nichols, & Smith, 2016; Winkler et al., 2014), enabling seamless integration with a wide array of alternative test statistics, including multivariate methods and TFCE (refer to Data and Code Availability section). Nonetheless, the integration of TCS with TFCE necessitates further research to design an efficient implementation, ascertain the optimal parameters for TFCE when combined with TCS, and comprehensively assess the added benefits arising from this combination of techniques.

### TCS Generalization: Alternative Topologies

The high-resolution anatomical connectivity structure is a fundamental part of TCS that could be tailored to a diverse set of study designs. In this study, we used a distance-dependent consensus high-resolution structural connectome to build a group-level anatomical network. This connectome is mainly constructed for normative group studies. Therefore, this network mainly consists of anatomical connections that were consistent in the whole group and can be used as a neuroanatomically meaningful basis for clustering group-level effects. It is, however, important to note that TCS can be used with other alternative connectomes and is not specific to the consensus connectome provided here.

With regard to alternative high-resolution connectomes, TCS could be generalized to other scenarios. For instance, an individual high-resolution connectome can be used to better capture fine anatomical detail. TCS can leverage this personalized connectome for individualized first-level analyses. Alternatively, a cohort-specific group-level connectome can be constructed to be tailored to a particular study cohort. In scenarios involving differences in brain connectivity between two cohorts, TCS can be implemented in two distinct settings. Firstly, TCS can be utilized via a healthy normative connectome. This allows TCS to infer group differences in activation with respect to a normative anatomical prior. This approach is particularly useful in cases where anatomical evidence, such as DWI data, may be lacking to directly measure connectivity. On the other hand, if connectivity information is available for each group, TCS can be applied separately within groups to identify significant clusters. Subsequently, the resulting clusters can be compared across groups, providing insights into functional brain changes as a consequence of connectivity alterations. It is important to note that these settings entail distinct implications that should be appropriately reflected in the resulting interpretations.

TCS could also be combined with connectomes built from neuroanatomical expert-informed tract atlases to further assist in the biological interpretation of activity maps (Catani & de Schotten, 2008; Wakana, Jiang, Nagae-Poetscher, van Zijl, & Mori, 2004; Zhang et al., 2018). Theoretically, TCS could even be adapted to use functional connectivity, thus enabling spatial clusters to form between functionally connected spatially disjoint networks. Importantly, if such alternative connectomes are to be considered, it is of utmost importance to validate connectome density and ensure adequate sparsity to avoid a less informative highly connected topology (see section on Anatomical Connectivity Structure for detail). The group-level consensus connectome that was used here is openly available, but additional example code is also provided to generate other alternative connectomes (see Data and Code Availability).

### Future Use

There are several ways that TCS can be used in future studies. For group-level inference, if a study has acquired diffusion data, then high-resolution connectomes computed from that data could be used to accurately cluster task fMRI activations. Nevertheless, if a study has only acquired functional neuroimaging data, an existing group-level connectome computed from high-quality public datasets (such as the one used in this study) can be exploited to localize activation networks. This latter scenario is based on an implicit assumption that the normative group-level anatomical backbone of the human brain is relatively consistent (controlling for covariates such as age or sex). Hence, assuming matched cohorts have comparable anatomical connections, neuroimaging data from a different cohort (with matching age/sex) can be used as the group-level anatomical connectivity to extract significant effects.

Similarly, for individualized studies, if high-resolution structural connectomes are available, they can be used to maximize the within-individual accuracy of white matter connections. Spatial normalization by connectome spatial smoothing must be implemented in such cases to increase the reliability of a single individual’s structural connectome (Mansour L. et al., 2022). If an individualized connectome is not available, a group-level connectome built from large-scale neuroimaging datasets, or white matter atlases, could similarly be exploited to approximate important white matter pathways shared across the population. The latter approach loses the accuracy benefits of individualized connectomes, but would enable TCS for cases where diffusion data was not acquired.

Finally, it should be noted that the interpretation benefits of TCS could even be applied to the results of prior cluster-based analyses. For instance, TCS could be applied to previously acquired group-level unthresholded statistics (such as those provided by NeuroSynth and NeuroVault (Gorgolewski et al., 2015, 2016) or to result of any other brain inference technique. This way, previously identified clusters of activation could be linked and related by the underlying structural connectivity to aid the interpretation of the spatially disjoint effects. While this use case does not benefit from the increased sensitivity of TCS, it can still gain data-driven insights for the interpretation of observed effects.

### Limitations

Several limitations require noting. First, the networks generated from TCS need to be interpreted with care. Anatomical connections between distributed clusters comprising a TCS map can potentially suggest that information is communicated via these connections between the clusters, to facilitate coordinated activity supporting task performance. It nevertheless does not infer edge-wise certainty or causality for the involved connections. In other words, TCS suggests that a subset of the suprathreshold network plays a role in the emerged activity. Hence, future work could integrate model-based approaches such as dynamic causal modeling (DCM) (Friston, Harrison, & Penny, 2003; Friston et al., 2019) with a prior model generated from TCS to assess any causal directed interactions between the anatomical edges.

Second, TCS uses information from diffusion and functional MRI and can consequently be affected by the limitations and biases of these modalities. For instance, TCS can be affected by the quality of the topological network structure used for clustering. In this paper, we used a state-of-the-art high-resolution group-level connectome to ensure the reliability of the anatomical topology (Mansour L., Tian, et al., 2021; Mansour L. et al., 2022). It is crucial to highlight that the quality and accuracy of connectome reconstruction can impact the performance of TCS. The provided scripts along with this manuscript aim to enable the extension of TCS to alternative connectome reconstruction approaches (Christiaens et al., 2015; Jeurissen, Descoteaux, Mori, & Leemans, 2019; Jeurissen, Tournier, Dhollander, Connelly, & Sijbers, 2014; Maier-Hein et al., 2017), including the use of individualized connectomes, while controlling for the quality of generated maps.

Finally, it is also crucial to highlight that TCS is specifically designed to enhance the identification of areally disjoint regions of significance that are interconnected by anatomical connections. Consequently, TCS may exhibit suboptimal performance in scenarios where the assumption of spatially disjoint, anatomically linked significant differences does not hold. Specifically, TCS may reduce sensitivity in detecting a hypothetical effect of interest that is localized to a single brain region. In such cases, the local effect cannot benefit from the enhancement provided by the anatomical topology, while competing nonparametric null effects may become more stringent. This phenomenon occurs due to the stochastic likelihood of spatially disjoint regions of null activation to be anatomically linked, resulting in a stronger null hypothesis.

### Concluding Remarks

In conclusion, we provide a novel cluster-based inference approach that utilizes anatomical connectivity priors extracted from high-resolution connectomes. Evidence from simulations and empirical data suggests that the method enhances the sensitivity and interpretability of cluster-based inferences and can better detect effects of a smaller spatial extent. Finally, compared to other cluster-based inference methods, TCS provides extra information that can be used to investigate the anatomical network implicated in an activation map.

## METHODS AND MATERIALS

### Imaging Data and Acquisition

Imaging data used in this work were obtained and made available by the Human Connectome Project (HCP) (Van Essen et al., 2013). We used task fMRI and diffusion MRI data from 1,000 individuals provided as part of the HCP S1200 release, available from https://db.humanconnectome.org/data/projects/HCP_1200. All imaging data were preprocessed according to HCP minimal preprocessing pipeline and no further preprocessing was undertaken (Glasser et al., 2013).

### Data Format

The imaging data was sourced in CIFTI format, which combines a surface representation of cortical regions with a volumetric representation of various subcortical regions including the cerebellum. This format contains a total of *N* = 91,282 brainordinates (Glasser et al., 2013) with 59,412 surface vertices situated over the fsLR-32k standard surface mesh and 31,870 volumetric voxels, all in a common geometry for all subjects. Brain activation maps and high-resolution connectivity matrices used this CIFTI structure. For visualization purposes, surfaces representing the 19 subcortical and cerebellar structures were generated. In anatomical network visualizations, these 19 subcortical and cerebellar structures were combined with the cortical regions from the Schaefer atlas (200 regions), grouped into seven resting-state networks (Schaefer et al., 2018; Yeo et al., 2011).

### Diffusion Data Processing

Probabilistic tractography was used to construct a normative group-level anatomical structure to guide task fMRI clustering with TCS. First, individual-level high-resolution structural connectivity matrices were produced with a probabilistic tractography pipeline (Mansour L. et al., 2022) using MRtrix3 software (Tournier et al., 2019). Specifically, the white matter (WM), gray matter (GM), and cerebrospinal fluid (CSF) response functions were estimated using an unsupervised method (Dhollander, Raffelt, & Connelly, 2016) followed by a constrained (Multi-Shell Multi-Tissue, MSMT) spherical deconvolution (Tournier, Calamante, Gadian, & Connelly, 2004) to estimate fiber orientation distribution (FOD) in each voxel. Probabilistic tractography by second-order integration over fiber orientation distributions (iFOD2) (Tournier, Calamante, & Connelly, 2010) was performed to generate five million streamlines for each individual. The streamline end points were subsequently warped to the MNI standard space and mapped to the closest CIFTI brainordinates. To improve anatomical plausibility, streamlines ending far from brainordinates (>2 mm) were discarded. The remaining streamlines, were used to generate subject-level high-resolution connectivity matrices *A*_*s*_ ∈ ℝ^*N*×*N*^ in which every element *A*_*s*_(*i*, *j*) indicated streamline count between brainordinates *i* and *j* (*s* ∈ {1, ·, 1,000} denotes an individual subject).

### Anatomical Connectivity Structure

Next, the individual structural connectivity matrices were combined to yield a group-level anatomical connectivity structure. To this end, we first calculated a group-level consensus matrix *C* ∈ ℝ^*N*×*N*^ whose elements indicated the frequency (consistency) of observing the connection across all individual connectomes:

$$C\left(i,j\right)=\frac{1}{1000}\sum_{s=1}^{1000}\left[A_{s}\left(i,j\right)>0\right]$$ (1)

This consistency index is inherently biased by the Euclidean distance of the connection end points such that short-range connections are generally more frequent (Betzel et al., 2019). To correct this bias, the Euclidean distance *ED* ∈ ℝ^*N*×*N*^ was computed for all observed connections. the consistency indices were discretely grouped into nonoverlapping bins based on *ED*. The values for mean (*μ*_*C*_ = *E*[*C*(*i*, *j*)]) and standard deviation (*σ*_*C*_ = $\sqrt{E\left[{\left(C\left(i,j\right)-\mu \right)}^{2}\right]}$) of consistencies within each *ED* bin was used to fit a nonlinear curve with cubic splines to model the variation of the consistency index as a function of distance:

$$\mu_{C}\left(i,j\right)=f_{\mu }\left(ED\left(i,j\right)\right)+\epsilon_{\mu }$$ (2)

$$\sigma_{C}\left(i,j\right)=f_{\sigma }\left(ED\left(i,j\right)\right)+\epsilon_{\sigma }$$ (3)

where *f*_*μ*_ and *f*_*σ*_ denote the fitted nonlinear estimates as a function of distance. A distance normalized consistency *C*_*n*_ was computed by subtracting the estimated mean from the original consistency index and dividing the result by the estimated standard deviation:

$$C_{n}\left(i,j\right)=\frac{C\left(i,j\right)-f_{\mu }\left(ED\left(i,j\right)\right)}{f_{\sigma }\left(ED\left(i,j\right)\right)}$$ (4)

This resulted in a consistency index *C*_*n*_, which is not biased by the distance between end points *ED*. This normalization is calculated independently for interhemispheric, intrahemispheric, and subcortical connections (Betzel et al., 2019). To increase the intersubject reliability and group-level comparability, the consistency matrix was smoothed using a 6 mm FWHM Gaussian smoothing with connectome spatial smoothing (CSS) (Mansour L., Seguin, Smith, & Zalesky, 2021; Mansour L. et al., 2022). The smoothed consistency matrix (*F*_*s*_*C*_*n*_$F_{s}^{T}$, where *F*_*s*_ denotes the smoothing kernel) was subsequently thresholded to maintain the average density of an individual connectome (Betzel et al., 2019; Roberts et al., 2017). Notably, the average density of *A*_*s*_ across all subjects was approximately 0.07%, that is, nearly three million binary high-resolution edges. This thresholding step generates a connectivity topology with a fixed density by selectively removing regions with lower evidence for the existence of a group-level anatomical connection. Finally, the thresholded smoothed distance-normalized consistency index was binarized to create a binary connectivity matrix *C*_*Dist*_ that is used by the TCS inference algorithm.

While we maintain connectome density by averaging individual network densities, as recommended previously (Betzel et al., 2019; Roberts et al., 2017), the density threshold serves as a tunable parameter influencing TCS performance. This threshold operates on a spectrum of values, impacting the resulting inference. A very high threshold reduces the topology to spatial rules, aligning TCS performance with conventional spatial cluster inferences. Conversely, an excessively low threshold results in a highly connected network, diminishing specificity and informedness in TCS performance as all suprathreshold regions form a single cluster. The optimal threshold lies somewhere between these extremes. While this perspective suggests the potential for tuning the threshold to maximize power, determining the optimal value can introduce complexity, especially considering its task-dependent nature. To maintain methodological consistency and avoid overcomplicating the TCS procedure, we opted for established approaches that determine a fixed threshold based on average individual connectome density.

It is also essential to highlight that if future studies plan to apply TCS with alternative topologies (as discussed in the section on TCS Generalization: Alternative Topologies), careful control and validation of the resulting network density are imperative. If the study is conducted within a comparable template space (i.e., fs-LR 32k), a straightforward approach is to use a density similar to this study (≤1%). Another viable option is to fix the density based on individual network densities of the alternative topology. The key consideration here is to ensure that the resultant topology maintains adequate sparsity, as highly connected topologies can significantly compromise the informativeness of TCS clusters.

### Spatial Structure

In addition to the anatomical connectivity structure derived from diffusion tractography, a spatial structure was constructed in the form of a binary connectivity matrix *S* ∈ ℝ^*N*×*N*^. For brainordinates on the surface mesh, nodes were connected to spatially adjacent nodes with a direct edge connection on the triangular mesh. For brainordinates on the volumetric space, nodes were connected to the 26-neighborhood nodes in their adjacent voxel grid.

### Anatomical Topology Structure

A topology *T* was constructed by combining the anatomical group structure *C*_*Dist*_ and the spatial structure *S* such that two brainordinates are considered to be topologically connected if there is either a spatial or an anatomical link between them:

$$T\left(i,j\right)=\left\{\begin{array}{l} 1\;\text{if}\;C_{\text{Dist}}\left(i,j\right)=1\;\text{or}\;S\left(i,j\right)=1\\ 0\;\text{otherwise} \end{array}\right.$$ (5)

### First-Level Task fMRI

First-level Contrast of Parameter Estimate (COPE) maps were sourced from HCP for consequent analyses. Five contrasts are selected from HCP’s preprocessed first-level task fMRI data (Barch et al., 2013) to enable direct comparison with earlier work (Noble et al., 2020). These contrasts encompass various potential activation patterns covering a range of local to spatially distributed effects with varying effect intensities and activation versus deactivation patterns. The tasks consisted of (i) the faces versus shapes contrast from the emotion task (EMOTION COPE 3; *N* = 988) with widespread (mainly positive) strong effects, (ii) the reward versus punishment contrast from the gambling task (GAMBLING COPE 6; *N* = 994) with widespread positive weak effects, (iii) the relational versus match contrast from the relational task (RELATIONAL COPE 4; *N* = 983) with widespread negative (deactivation) and localized positive moderate effects, (iv) the theory of mind versus random contrast from the social task (SOCIAL COPE 6; *N* = 991), and (v) the face versus other contrast from the working memory task (WM COPE 20; *N* = 992) with localized negative strong effects and moderate positive effects. First-level surface-based (CIFTI) statistics were sourced for every contrast of parameter estimate (COPE) explained above.

### Putative Ground Truth

To quantify statistical power and specificity, an initial ground truth estimate was required. However, finding the realistic ground truth activation map of a fixed task contrast can be challenging. Here, we implemented a previously established approach to construct a putative ground truth based on a large enough sample (Cremers et al., 2017; Noble et al., 2020). From a statistical standpoint, the effect observed in a sample should converge to the ground truth given a sufficiently large sample size. We aimed to evaluate the performance of different inference methods in capturing the effect at a large sample size given a limited sample. Thus, we used the effect observed across the entire HCP sample to form a putative ground truth. For each task, ground truth effect sizes were estimated from a group-level one-sample *t* statistic computed at the level of brainordinates. To provide a sample-size-independent measure of effect size, the *t* statistics were then converted to Cohen’s *d* coefficients (*d* = $\frac{t}{\sqrt{N}}$) (Cohen, 1988). This provided putative ground truth task activation maps observed in a comparatively larger sample size than what is normally practiced in neuroimaging studies (Szucs & Ioannidis, 2020; Yeung, 2018). Notably, this definition of the putative ground truth is reportedly robust against sign errors (Noble et al., 2022) and can thus evaluate the correctness of inferred effect signs.

### Statistical Power

While traditional cluster-based spatial statistic (Friston et al., 1994) has recognized limitations (Eklund et al., 2016; Noble et al., 2020, 2022) that have prompted the development of various enhancement techniques (Bowring, Telschow, Schwartzman, & Nichols, 2021; Geerligs & Maris, 2021; Smith & Nichols, 2009; Winkler et al., 2014), it continues to be the prevailing method in studying task activation (Acar et al., 2023; Carp, 2012; Woo, Krishnan, & Wager, 2014) and retains popularity across various study domains (Cook et al., 2020; Jauhar et al., 2021; Lee et al., 2023; Tannou, Magnin, Comte, Aubry, & Joubert, 2021). In lack of previous methods that integrate anatomical connectivity information to enhance inference sensitivity, we compared the performance of our method with this commonly used alternative for task inferences (Acar et al., 2023; Carp, 2012), that is, the spatial cluster–based statistic (Friston et al., 1994). This comparison was made based on the sensitivity in detecting true effects across different sample sizes. We used a conventional empirical benchmarking method to evaluate statistical power (Noble et al., 2020).

Namely, for a total of 500 repetitions, a randomly selected subsample with a fixed size (*N* = 40) was drawn from the whole sample (supplemental replications were performed for *N* ∈ {10, 20, 40, 80, 160, 320}). For each subsample, a one-sample two-sided cluster-based inference with nonparametric FWER correction for multiple testing was conducted using either TCS or spatial cluster-extent statistic. More specifically, a group-level statistical map was computed using a one-sample two-sided *t* statistic; The group-level statistical map was thresholded with a stringent cluster-defining threshold (*z* = 3.3 which corresponds to *p* = 0.001 for a two-sided test) to delineate suprathreshold activations (Woo et al., 2014) (supplemental replications were also performed for *z* ∈ {3.3, 2.8, 2.6, 2.0, 1.6}); thereafter, a connectivity structure was used to cluster the suprathreshold regions into activation components.

The spatial cluster–based method used the spatial structure *S*, whereas TCS used the topological structure *T*. In both approaches, a nonparametric permutation test with sign flipping was used to provide a *p* value for all activation components (1,000 permutations) (Winkler et al., 2014). This approach was specifically adopted to address recognized limitations of parametric statistical methods, as highlighted in prior studies (Eklund et al., 2016). Utilizing this nonparametric implementation mitigates this issue and ensures nominal family-wise error control through a permutation test. An FWER corrected *p* value threshold of 0.05 was used to mark significant activation at the level of clusters. This yielded two binary maps of brainordinates that survived correction over competing respective structures (spatial or TCS) for each repetition.

Next, for every brainordinate, statistical power was computed relative to the ground truth effect observed at that brainordinate. Statistical power was calculated as the proportion of random repetitions in which the brainordinate effect was correctly detected in a significant activation cluster (with the same activation sign as the ground truth). Then, a moving window average was used to measure average statistical power at different effect sizes. A cubic spline was fitted to the moving average to provide a continuous estimate of statistical power as a function of effect size. Additionally, the absolute differences in true positive rate curves were reported to illustrate the benefits of TCS as a function of ground truth effect size. This test was similarly repeated at different sample sizes to capture the relative importance of sample size.

Additionally, a power analysis was conducted to quantify the range of possible sensitivity values. The power analyses evaluated the power of a one-sample *t* test without any corrections, with a test level *α* < 0.05 (sensitivity upper bound). Similarly, another power analysis evaluated sensitivity after a Bonferroni correction over all brainordinates, *α* < $\frac{0.05}{91282}$, giving a lower bound for sensitivity. The TTestPower function from the statmodels module (in Python) was used for power calculation. The results of these power analyses are depicted as two sets of dashed lines in Figure 3, Supporting Information Figures S1, and S2.

### Classifier Informedness

The outcome of a cluster inference analysis could abstractly be viewed as a binary classification in which brainordinates are classified into significant and nonsignificant regions. Statistical power indicates the sensitivity at detecting the true significant results (true positive rate). Nevertheless, while a higher true positive rate is ideal, we need to ensure that it does not impose significant reductions in specificity (true negative rate). We hence computed a measure of classification performance (the bookmaker informedness index, *BM*) to evaluate the overall classification improvement. *BM* was selected as it provided an unbiased measure of classification performance compared to other metrics (i.e., accuracy or F1-score).

To evaluate classification performance, the putative ground truth was discretized to (i) positive significant, (ii) negative significant, and (iii) null regions. Hence, a threshold on Cohen’s *d* was used for this discretization, as such null regions constituted less meaningful effects (Sullivan & Feinn, 2012). Next, for each random repetition, the number of brainordinates in significant regions that correctly survived inference correction indicated the true positives *TP*; and the remaining brainordinates, which were incorrectly inferred as significant formed the false positives *FP*. Similarly, among brainordinates that were not part of any significant active cluster, those that were correctly classified according to the ground truth formed true negatives *TN*, and the remaining brainordinates were considered false negatives *FN*. The true positive rate *TPR* = $\frac{TP}{TP+FN}$ indicated the proportion of truly active brainordinates that were correctly classified, whereas the true negative rate *TNR* = $\frac{TN}{TN+FP}$ marked the proportion of correctly classified truly inactive brainordinates. The informedness could thus be defined as follows:

BM=TPR+TNR−1 (6)

This value ranges from −1 to 1, and higher scores indicate more informed classifications. Importantly, the expected value for the informedness of a random classifier is zero, and positive values indicate classifications that are more informed than chance level.

### Binarization Threshold Range

Classification performance quantification required a fixed binarization threshold to discretize the putative ground truth maps into a binary set of significant versus null effects. To ensure that our comparisons were not specific to a particular binarization threshold, we evaluated classification performance for a range of possible binarization thresholds (from ∣*d*∣ = 0.06 to ∣*d*∣ = 0.16). This selection spans a reasonable range of thresholds for the ground truth because it includes (i) the effect size that is detectable using an uncorrected *p* value threshold of 0.05 (∣*d*∣ ≃ 0.06) and (ii) the effect size that survives a Bonferroni correction of multiple comparisons (at the level of brainordinates) with a 5% confidence (both thresholds are for a sample of *N* = 1,000). Hence, this threshold range (0.06 ≤ ∣*d*∣ ≤ 0.16) spans from the most liberal to the most stringent choices of an effect threshold for the putative ground truth. Informedness evaluations were repeated along this range of feasible thresholds and an aggregate measure of the normalized partial area under the curve (confined to the threshold range) was used to summarize the comparisons into a threshold-independent informedness metric.

### Alternative Evaluations

We additionally conducted alternative evaluations of inference performance that further validated the main findings. For the sake of brevity, the Supporting Information includes a detailed presentation of these evaluations. Namely, a different assessment of sensitivity and specificity was conducted by computing the normalized partial area under the receiver operating characteristic curve (see Supporting Information Figure S9). Furthermore, a quantification of the inference success ratio defined by the likelihood of detecting any significant clusters was provided (see Supporting Information Figure S10).

### Network Visualizations

Three different visualization approaches were presented to summarize the high-resolution anatomical network implicated in a task activation map, namely: (i) a connectogram, (ii) a spatially confined network view, and (iii) a summarized tree of most likely implicated connections. For all visualizations, the high-resolution binary anatomical connectivity matrix *C*_*Dist*_ was first filtered to only keep connections between brainordinates that survive correction; the remaining connections were deemed to be not directly involved in the effect and were hence set to zero. This filtered high-resolution anatomical connectivity matrix was used for network visualizations.

For the connectogram, this high-resolution matrix was downsampled to the resolution of 200 cortical regions (Schaefer et al., 2018) and 19 volumetric structures comprising subcortical nuclei, the cerebellum, and the brain stem. Specifically, a binary connectivity matrix was generated where the edge weight between two regions was set to one if any high-resolution edge connected the two regions. A left-right symmetric chord diagram view was generated to show this downsampled anatomical network visualization (using the pyCircos Python module available at github.com/ponnhide/pyCircos). The left cortex, right cortex, and volumetric regions were separated to aid interpretation. Additionally, cortical regions were color-coded based on their respective functional resting-state networks (Yeo et al., 2011).

Next, for the 3D network visualizations, the brainordinates that survived correction were filtered to keep spatially contingent clusters that were at least 1% of the size of the largest detected spatial cluster (size was quantified by the number of brainordinates in a cluster). This masked the spatially minuscule effects and enabled focusing on the connectivity between larger clusters. The downsampled anatomical connectivity between these larger spatially disjoint clusters was visualized using a ball and stick visualization. Furthermore, another 3D visualization was made to probe the most likely edges within this network. To this end, we assigned weights to all anatomical edges connecting spatially disjoint clusters. For every edge, the assigned weight was the multiplication of the maximum effect size observed at each respective spatial cluster connected by that edge. This weighted network was then filtered using a maximum spanning tree algorithm (Kruskal’s algorithm). The resulting filtered network presented a minimally connected subset of the anatomical network that was most strongly implicated in the task.

### Simulated Data

Apart from the empirical data, a hypothetical simulation was used to present the benefits of TCS compared to spatial cluster–based inference. An 80 × 80 square grid, representing an axial image slice, was masked by a circular ribbon. This ribbon was further divided into 11 hypothetical atlas-delineated areas (five representing each hemisphere and one representing the brain stem). A ground truth signal spanning three regions was added to this ribbon. In this ground truth, active pixels had a peak of 1 and a background value of 0. Gaussian white noise with a standard deviation of 2 was added to the original signal and spatially smoothed (*σ* = 0.5) to represent a signal with noise contamination. A cluster-defining threshold of 2 was used to threshold pixels for clustering.

Thereafter, TCS and spatial cluster-extent methods were used to cluster active regions. For spatial clustering, a four neighborhood lattice structure was used to define the two-dimensional spatial neighborhood of every pixel to its surrounding pixels. For TCS, a set of long-range connections was added to the spatial structure. These were added to represent hypothetical white matter connections and thus included (i) short-range connections between neighboring regions resembling short-range association fibers, (ii) long-range connections originating from the brain stem region representing projection fibers, and (iii) homotopic connections between the left and right regions representing commissural fibers.

## ACKNOWLEDGMENTS

Data were provided by the Human Connectome Project, WU-Minn Consortium (Principal Investigators: David Van Essen and Kamil Ugurbil; 1U54MH091657) funded by the 16 NIH Institutes and Centers that support the NIH Blueprint for Neuroscience Research; and by the McDonnell Center for Systems Neuroscience at Washington University. The data analysis was supported by SPARTAN High-Performance Computing System at the University of Melbourne (Meade, Lafayette, Sauter, & Tosello, 2017), and also supported by the use of the Melbourne Research Cloud (MRC) providing Infrastructure-as-a-Service (IaaS) cloud computing to the University of Melbourne researchers through the NeCTAR Research Cloud, a collaborative Australian research platform supported by the National Collaborative Research Infrastructure Strategy.

## SUPPORTING INFORMATION

Supporting information for this article is available at https://doi.org/10.1162/netn_a_00375.

## AUTHOR CONTRIBUTIONS

Sina Mansour L.: Conceptualization; Formal analysis; Investigation; Methodology; Software; Visualization; Writing – original draft; Writing – review & editing. Caio Seguin: Conceptualization; Supervision; Writing – original draft; Writing – review & editing. Anderson Winkler: Formal analysis; Software; Writing – original draft; Writing – review & editing. Stephanie Noble: Writing – original draft; Writing – review & editing. Andrew Zalesky: Conceptualization; Funding acquisition; Supervision; Writing – original draft; Writing – review & editing.

## FUNDING INFORMATION

Andrew Zalesky, National Health and Medical Research Council (https://dx.doi.org/10.13039/501100000925), Award ID: APP1118153. Sina Mansour L., The University of Melbourne, Award ID: Melbourne Research Scholarship (319357).

## DATA AND CODE AVAILABILITY

All imaging data used to conduct this study is available from the Human Connectome Project (HCP) (www.humanconnectome.org). The computations required to perform the cluster inference methods are available in the Permutation Analysis of Linear Models (PALM) tool (Winkler et al., 2014, 2016), available from the FSL website at fsl.fmrib.ox.ac.uk/fsl/fslwiki/PALM. Additional scripts used for visualization and interpretation were mainly written in Python 3 and use several open-source packages including SciPy (Virtanen et al., 2020), Nibabel (Brett et al., 2020), CSS (Mansour L., Tian, et al., 2021), and Cerebro (Mansour L., 2023). Example Python scripts and accompanying data required to replicate the analyses or perform TCS are made openly available to facilitate future research and promote open transparent practices in code-sharing (Gilmore, Diaz, Wyble, & Yarkoni, 2017; Gleeson, Davison, Silver, & Ascoli, 2017; Smout et al., 2021). The supplementary code and data are available in a GitHub repository hosted at github.com/sina-mansour/Topological_Cluster_Statistic. The repository also hosts mapped group-level topological connectomes and example scripts to generate alternative topological structures for TCS, as well as high-resolution versions of all images and plots included in the manuscript.

## Supplementary Material



## TECHNICAL TERMS

Structural connectivity: The network of anatomical connections between different brain regions, typically generated from diffusion MRI tractography.
High-resolution connectomics: Detailed mapping and analysis of brain connectivity at the level of voxels and vertices.
Sensitivity: Also referred to as power, sensitivity represents the likelihood that a study will detect an effect when it truly exists, minimizing false negatives.
Specificity: The ability of a test to correctly identify instances where the null hypothesis is true, minimizing false positives.
Brainordinate: A precise location within the brain identified by a vertex on a surface mesh or a voxel in a volumetric grid.

## References

[bib1] Acar, F., Maumet, C., Heuten, T., Vervoort, M., Bossier, H., Seurinck, R., & Moerkerke, B. (2023). Review paper: Reporting practices for task fMRI studies. Neuroinformatics, 21(1), 221–242. 10.1007/s12021-022-09606-2, 36199009

[bib2] Barch, D. M., Burgess, G. C., Harms, M. P., Petersen, S. E., Schlaggar, B. L., Corbetta, M., … WU-Minn HCP Consortium. (2013). Function in the human connectome: Task-fMRI and individual differences in behavior. NeuroImage, 80, 169–189. 10.1016/j.neuroimage.2013.05.033, 23684877 PMC4011498

[bib3] Baum, G. L., Cui, Z., Roalf, D. R., Ciric, R., Betzel, R. F., Larsen, B., … Satterthwaite, T. D. (2020). Development of structure–function coupling in human brain networks during youth. Proceedings of the National Academy of Sciences, 117(1), 771–778. 10.1073/pnas.1912034117, 31874926 PMC6955327

[bib4] Betzel, R. F., Griffa, A., Hagmann, P., & Mišić, B. (2019). Distance-dependent consensus thresholds for generating group-representative structural brain networks. Network Neuroscience, 3(2), 475–496. 10.1162/netn_a_00075, 30984903 PMC6444521

[bib5] Bowring, A., Telschow, F. J., Schwartzman, A., & Nichols, T. E. (2021). Confidence sets for Cohen’s *d* effect size images. NeuroImage, 226, 117477. 10.1016/j.neuroimage.2020.117477, 33166643 PMC7836238

[bib6] Brett, M., Markiewicz, C. J., Hanke, M., Côté, M.-A., Cipollini, B., McCarthy, P., … Freec84. (2020). nipy/nibabel. Zenodo. 10.5281/zenodo.591597

[bib7] Bullmore, E. T., Suckling, J., Overmeyer, S., Rabe-Hesketh, S., Taylor, E., & Brammer, M. J. (1999). Global, voxel, and cluster tests, by theory and permutation, for a difference between two groups of structural mr images of the brain. IEEE Transactions on Medical Imaging, 18(1), 32–42. 10.1109/42.750253, 10193695

[bib8] Carp, J. (2012). The secret lives of experiments: Methods reporting in the fMRI literature. NeuroImage, 63(1), 289–300. 10.1016/j.neuroimage.2012.07.004, 22796459

[bib9] Catani, M., & de Schotten, M. T. (2008). A diffusion tensor imaging tractography atlas for virtual in vivo dissections. Cortex, 44(8), 1105–1132. 10.1016/j.cortex.2008.05.004, 18619589

[bib10] Christiaens, D., Reisert, M., Dhollander, T., Sunaert, S., Suetens, P., & Maes, F. (2015). Global tractography of multi-shell diffusion-weighted imaging data using a multi-tissue model. NeuroImage, 123, 89–101. 10.1016/j.neuroimage.2015.08.008, 26272729

[bib11] Cohen, J. (1988). Statistical power analysis for the behavioral sciences (2nd ed.). Lawrence Erlbaum Associates. 10.4324/9780203771587

[bib12] Cook, M. J., Gardner, A. J., Wojtowicz, M., Williams, W. H., Iverson, G. L., & Stanwell, P. (2020). Task-related functional magnetic resonance imaging activations in patients with acute and subacute mild traumatic brain injury: A coordinate-based meta-analysis. NeuroImage: Clinical, 25, 102129. 10.1016/j.nicl.2019.102129, 31891819 PMC6939096

[bib13] Cremers, H. R., Wager, T. D., & Yarkoni, T. (2017). The relation between statistical power and inference in fMRI. PLoS One, 12(11), 1–20. 10.1371/journal.pone.0184923, 29155843 PMC5695788

[bib14] Dhollander, T., Raffelt, D., & Connelly, A. (2016). Unsupervised 3-tissue response function estimation from single-shell or multi-shell diffusion MR data without a co-registered T1 image. In ISMRM workshop on breaking the barriers of diffusion MRI (p. 5). https://www.researchgate.net/publication/307863133_Unsupervised_3-tissue_response_function_estimation_from_single-shell_or_multi-shell_diffusion_MR_data_without_a_co-registered_T1_image

[bib15] Eklund, A., Nichols, T. E., & Knutsson, H. (2016). Cluster failure: Why fMRI inferences for spatial extent have inflated false-positive rates. Proceedings of the National Academy of Sciences of the United States of America, 113(28), 7900–7905. 10.1073/pnas.1602413113, 27357684 PMC4948312

[bib16] Forman, S. D., Cohen, J. D., Fitzgerald, M., Eddy, W. F., Mintun, M. A., & Noll, D. C. (1995). Improved assessment of significant activation in functional magnetic resonance imaging (fMRI): Use of a cluster-size threshold. Magnetic Resonance in Medicine, 33(5), 636–647. 10.1002/mrm.1910330508, 7596267

[bib17] Fornito, A., Zalesky, A., & Breakspear, M. (2013). Graph analysis of the human connectome: Promise, progress, and pitfalls. NeuroImage, 80, 426–444. 10.1016/j.neuroimage.2013.04.087, 23643999

[bib18] Friston, K. J., Harrison, L., & Penny, W. (2003). Dynamic causal modelling. NeuroImage, 19(4), 1273–1302. 10.1016/S1053-8119(03)00202-7, 12948688

[bib19] Friston, K. J., Preller, K. H., Mathys, C., Cagnan, H., Heinzle, J., Razi, A., & Zeidman, P. (2019). Dynamic causal modelling revisited. NeuroImage, 199, 730–744. 10.1016/j.neuroimage.2017.02.045, 28219774 PMC6693530

[bib20] Friston, K. J., Worsley, K. J., Frackowiak, R. S. J., Mazziotta, J. C., & Evans, A. C. (1994). Assessing the significance of focal activations using their spatial extent. Human Brain Mapping, 1(3), 210–220. 10.1002/hbm.460010306, 24578041

[bib21] Geerligs, L., & Maris, E. (2021). Improving the sensitivity of cluster-based statistics for functional magnetic resonance imaging data. Human Brain Mapping, 42(9), 2746–2765. 10.1002/hbm.25399, 33724597 PMC8127161

[bib22] Gilmore, R. O., Diaz, M. T., Wyble, B. A., & Yarkoni, T. (2017). Progress toward openness, transparency, and reproducibility in cognitive neuroscience. Annals of the New York Academy of Sciences, 1396(1), 5–18. 10.1111/nyas.13325, 28464561 PMC5545750

[bib23] Glasser, M. F., Sotiropoulos, S. N., Wilson, J. A., Coalson, T. S., Fischl, B., Andersson, J. L., … WU-Minn HCP Consortium. (2013). The minimal preprocessing pipelines for the Human Connectome Project. NeuroImage, 80, 105–124. 10.1016/j.neuroimage.2013.04.127, 23668970 PMC3720813

[bib24] Gleeson, P., Davison, A. P., Silver, R. A., & Ascoli, G. A. (2017). A commitment to open source in neuroscience. Neuron, 96(5), 964–965. 10.1016/j.neuron.2017.10.013, 29216458

[bib25] Glover, G. H. (2011). Overview of functional magnetic resonance imaging. Neurosurgery Clinics of North America, 22(2), 133–139. 10.1016/j.nec.2010.11.001, 21435566 PMC3073717

[bib27] Gorgolewski, K. J., Varoquaux, G., Rivera, G., Schwarz, Y., Ghosh, S. S., Maumet, C., … Margulies, D. S. (2015). NeuroVault.org: A web-based repository for collecting and sharing unthresholded statistical maps of the human brain. Frontiers in Neuroinformatics, 9, 8. 10.3389/fninf.2015.00008, 25914639 PMC4392315

[bib26] Gorgolewski, K. J., Varoquaux, G., Rivera, G., Schwartz, Y., Sochat, V. V., Ghosh, S. S., … Poldrack, R. A. (2016). NeuroVault.org: A repository for sharing unthresholded statistical maps, parcellations, and atlases of the human brain. NeuroImage, 124, 1242–1244. 10.1016/j.neuroimage.2015.04.016, 25869863 PMC4806527

[bib28] Hadj-Bouziane, F., Liu, N., Bell, A. H., Gothard, K. M., Luh, W. M., Tootell, R. B., … Ungerleider, L. G. (2012). Amygdala lesions disrupt modulation of functional MRI activity evoked by facial expression in the monkey inferior temporal cortex. Proceedings of the National Academy of Sciences of the United States of America, 109(52), E3640–E3648. 10.1073/pnas.1218406109, 23184972 PMC3535608

[bib29] Hagmann, P., Cammoun, L., Gigandet, X., Meuli, R., Honey, C. J., Wedeen, V. J., & Sporns, O. (2008). Mapping the structural core of human cerebral cortex. PLoS Biology, 6(7), e159. 10.1371/journal.pbio.0060159, 18597554 PMC2443193

[bib30] Haxby, J. V., Hoffman, E. A., & Gobbini, M. (2000). The distributed human neural system for face perception. Trends in Cognitive Sciences, 4(6), 223–233. 10.1016/S1364-6613(00)01482-0, 10827445

[bib31] Heeger, D. J., & Ress, D. (2002). What does fMRI tell us about neuronal activity? Nature Reviews Neuroscience, 3(2), 142–151. 10.1038/nrn730, 11836522

[bib32] Honey, C. J., Sporns, O., Cammoun, L., Gigandet, X., Thiran, J. P., Meuli, R., & Hagmann, P. (2009). Predicting human resting-state functional connectivity from structural connectivity. Proceedings of the National Academy of Sciences of the United States of America, 106(6), 2035–2040. 10.1073/pnas.0811168106, 19188601 PMC2634800

[bib33] Jauhar, S., Fortea, L., Solanes, A., Albajes-Eizagirre, A., McKenna, P. J., & Radua, J. (2021). Brain activations associated with anticipation and delivery of monetary reward: A systematic review and meta-analysis of fMRI studies. PLoS ONE, 16(8), 1–21. 10.1371/journal.pone.0255292, 34351957 PMC8341642

[bib34] Jeurissen, B., Descoteaux, M., Mori, S., & Leemans, A. (2019). Diffusion MRI fiber tractography of the brain. NMR in Biomedicine, 32(4), e3785. 10.1002/nbm.3785, 28945294

[bib35] Jeurissen, B., Tournier, J. D., Dhollander, T., Connelly, A., & Sijbers, J. (2014). Multi-tissue constrained spherical deconvolution for improved analysis of multi-shell diffusion MRI data. NeuroImage, 103, 411–426. 10.1016/j.neuroimage.2014.07.061, 25109526

[bib36] Lee, K. S., Hagan, C. N., Hughes, M., Cotter, G., McAdam Freud, E., Kircanski, K., … Tseng, W.-L. (2023). Systematic review and meta-analysis: Task-based fMRI studies in youths with irritability. Journal of the American Academy of Child and Adolescent Psychiatry, 62(2), 208–229. 10.1016/j.jaac.2022.05.014, 35944754 PMC9892288

[bib37] Leonard, C. M., Rolls, E. T., Wilson, F. A., & Baylis, G. C. (1985). Neurons in the amygdala of the monkey with responses selective for faces. Behavioural Brain Research, 15(2), 159–176. 10.1016/0166-4328(85)90062-2, 3994832

[bib38] Liu, Z.-Q., Vázquez-Rodríguez, B., Spreng, R. N., Bernhardt, B. C., Betzel, R. F., & Misic, B. (2022). Time-resolved structure-function coupling in brain networks. Communications Biology, 5(1), 1–10. 10.1038/s42003-022-03466-x, 35654886 PMC9163085

[bib39] Logothetis, N. K. (2008). What we can do and what we cannot do with fMRI. Nature, 453(7197), 869–878. 10.1038/nature06976, 18548064

[bib40] Lohmann, G., Stelzer, J., Lacosse, E., Kumar, V. J., Mueller, K., Kuehn, E., … Scheffler, K. (2018). LISA improves statistical analysis for fMRI. Nature Communications, 9(1), 1–9. 10.1038/s41467-018-06304-z, 30275541 PMC6167367

[bib41] Luque, A., Carrasco, A., Martín, A., & de las Heras, A. (2019). The impact of class imbalance in classification performance metrics based on the binary confusion matrix. Pattern Recognition, 91, 216–231. 10.1016/j.patcog.2019.02.023

[bib42] Maier-Hein, K. H., Neher, P. F., Houde, J.-C., Côté, M.-A., Garyfallidis, E., Zhong, J., … Descoteaux, M. (2017). The challenge of mapping the human connectome based on diffusion tractography. Nature Communications, 8(1), 1349. 10.1038/s41467-017-01285-x, 29116093 PMC5677006

[bib43] Mansour L., S. (2023). Cerebro_Viewer A Pythonic 3D viewer to visualize and plot brains (v0.0.9). Zenodo. 10.5281/zenodo.7885669

[bib44] Mansour L., S., Seguin, C., Smith, R. E., & Zalesky, A. (2021). Connectome spatial smoothing v.0.1.1. Zenodo. 10.5281/zenodo.5746619 35077853

[bib45] Mansour L., S., Seguin, C., Smith, R. E., & Zalesky, A. (2022). Connectome spatial smoothing (CSS): Concepts, methods, and evaluation. NeuroImage, 250, 118930. 10.1016/j.neuroimage.2022.118930, 35077853

[bib46] Mansour L., S., Tian, Y., Yeo, B. T. T., Cropley, V., & Zalesky, A. (2021). High-resolution connectomic fingerprints: Mapping neural identity and behavior. NeuroImage, 229, 117695. 10.1016/j.neuroimage.2020.117695, 33422711

[bib47] McDonald, A. J. (1998). Cortical pathways to the mammalian amygdala. Progress in Neurobiology, 55(3), 257–332. 10.1016/S0301-0082(98)00003-3, 9643556

[bib48] McFadyen, J., Mermillod, M., Mattingley, J. B., Halász, V., & Garrido, M. I. (2017). A rapid subcortical amygdala route for faces irrespective of spatial frequency and emotion. Journal of Neuroscience, 37(14), 3864–3874. 10.1523/JNEUROSCI.3525-16.2017, 28283563 PMC6596715

[bib49] Meade, B., Lafayette, L., Sauter, G., & Tosello, D. (2017). Spartan HPC-Cloud Hybrid: Delivering performance and flexibility. University of Melbourne. Online resource. 10.4225/49/58ead90dceaaa

[bib50] Mumford, J. A., & Nichols, T. E. (2008). Power calculation for group fMRI studies accounting for arbitrary design and temporal autocorrelation. NeuroImage, 39(1), 261–268. 10.1016/j.neuroimage.2007.07.061, 17919925 PMC2423281

[bib51] Nichols, T. E., & Holmes, A. P. (2002). Nonparametric permutation tests for functional neuroimaging: A primer with examples. Human Brain Mapping, 15(1), 1–25. 10.1002/hbm.1058, 11747097 PMC6871862

[bib52] Noble, S., Mejia, A. F., Zalesky, A., & Scheinost, D. (2022). Improving power in functional magnetic resonance imaging by moving beyond cluster-level inference. Proceedings of the National Academy of Sciences, 119(32), 1–10. 10.1073/pnas.2203020119, 35925887 PMC9371642

[bib53] Noble, S., Scheinost, D., & Constable, R. T. (2020). Cluster failure or power failure? Evaluating sensitivity in cluster-level inference. NeuroImage, 209, 116468. 10.1016/j.neuroimage.2019.116468, 31852625 PMC8061745

[bib54] Pessoa, L., & Adolphs, R. (2010). Emotion processing and the amygdala: From a ‘low road’ to ‘many roads’ of evaluating biological significance. Nature Reviews Neuroscience, 11(11), 773–782. 10.1038/nrn2920, 20959860 PMC3025529

[bib55] Poldrack, R. A., Baker, C. I., Durnez, J., Gorgolewski, K. J., Matthews, P. M., Munafò, M. R., … Yarkoni, T. (2017). Scanning the horizon: Towards transparent and reproducible neuroimaging research. Nature Reviews Neuroscience, 18(2), 115–126. 10.1038/nrn.2016.167, 28053326 PMC6910649

[bib56] Powers, D. M. W. (2020). Evaluation: From precision, recall and F-measure to ROC, informedness, markedness and correlation. arXiv. 10.48550/arXiv.2010.16061

[bib57] Roberts, J. A., Perry, A., Roberts, G., Mitchell, P. B., & Breakspear, M. (2017). Consistency-based thresholding of the human connectome. NeuroImage, 145, 118–129. 10.1016/j.neuroimage.2016.09.053, 27666386

[bib58] Sarwar, T., Tian, Y., Yeo, B. T. T., Ramamohanarao, K., & Zalesky, A. (2021). Structure-function coupling in the human connectome: A machine learning approach. NeuroImage, 226, 117609. 10.1016/j.neuroimage.2020.117609, 33271268

[bib59] Schaefer, A., Kong, R., Gordon, E. M., Laumann, T. O., Zuo, X.-N., Holmes, A. J., … Yeo, B. T. T. (2018). Local-global parcellation of the human cerebral cortex from intrinsic functional connectivity MRI. Cerebral Cortex, 28(9), 3095–3114. 10.1093/cercor/bhx179, 28981612 PMC6095216

[bib60] Seguin, C., Mansour L., S., Sporns, O., Zalesky, A., & Calamante, F. (2022). Network communication models narrow the gap between the modular organization of structural and functional brain networks. NeuroImage, 257, 119323. 10.1016/j.neuroimage.2022.119323, 35605765

[bib61] Smith, S. M., & Nichols, T. E. (2009). Threshold-free cluster enhancement: Addressing problems of smoothing, threshold dependence and localisation in cluster inference. NeuroImage, 44(1), 83–98. 10.1016/j.neuroimage.2008.03.061, 18501637

[bib62] Smout, C., Holford, D. L., Garner, K., Illanes, M., Martinez, P. A., Campbell, M. E. J., … Coelho, L. P. (2021). An open code pledge for the neuroscience community. MetaArXiv. 10.31222/osf.io/vrwm7

[bib63] Sullivan, G. M., & Feinn, R. (2012). Using effect size—Or why the *p* value is not enough. Journal of Graduate Medical Education, 4(3), 279–282. 10.4300/JGME-D-12-00156.1, 23997866 PMC3444174

[bib64] Szucs, D., & Ioannidis, J. P. (2020). Sample size evolution in neuroimaging research: An evaluation of highly-cited studies (1990–2012) and of latest practices (2017–2018) in high-impact journals. NeuroImage, 221, 117164. 10.1016/j.neuroimage.2020.117164, 32679253

[bib65] Tannou, T., Magnin, E., Comte, A., Aubry, R., & Joubert, S. (2021). Neural activation in risky decision-making tasks in healthy older adults: A meta-analysis of fMRI data. Brain Sciences, 11(8), 1043. 10.3390/brainsci11081043, 34439662 PMC8393360

[bib66] Tournier, J.-D., Calamante, F., & Connelly, A. (2010). Improved probabilistic streamlines tractography by 2nd order integration over fibre orientation distributions. ISMRM, 18, 1670.

[bib67] Tournier, J.-D., Calamante, F., Gadian, D. G., & Connelly, A. (2004). Direct estimation of the fiber orientation density function from diffusion-weighted MRI data using spherical deconvolution. NeuroImage, 23(3), 1176–1185. 10.1016/j.neuroimage.2004.07.037, 15528117

[bib68] Tournier, J.-D., Smith, R., Raffelt, D., Tabbara, R., Dhollander, T., Pietsch, M., … Connelly, A. (2019). MRtrix3: A fast, flexible and open software framework for medical image processing and visualisation. NeuroImage, 202, 116137. 10.1016/j.neuroimage.2019.116137, 31473352

[bib69] Tsao, D. Y., & Livingstone, M. S. (2008). Mechanisms of face perception. Annual Review of Neuroscience, 31, 411–437. 10.1146/annurev.neuro.30.051606.094238, 18558862 PMC2629401

[bib70] Turner, B. O., Paul, E. J., Miller, M. B., & Barbey, A. K. (2018). Small sample sizes reduce the replicability of task-based fMRI studies. Communications Biology, 1(1), 62. 10.1038/s42003-018-0073-z, 30271944 PMC6123695

[bib71] van den Heuvel, M. P., Mandl, R. C. W., Kahn, R. S., & Hulshoff Pol, H. E. (2009). Functionally linked resting-state networks reflect the underlying structural connectivity architecture of the human brain. Human Brain Mapping, 30(10), 3127–3141. 10.1002/hbm.20737, 19235882 PMC6870902

[bib72] Van Essen, D. C., Smith, S. M., Barch, D. M., Behrens, T. E. J., Yacoub, E., Ugurbil, K., & WU-Minn HCP Consortium. (2013). The WU-Minn Human Connectome Project: An overview. NeuroImage, 80, 62–79. 10.1016/j.neuroimage.2013.05.041, 23684880 PMC3724347

[bib73] Virtanen, P., Gommers, R., Oliphant, T. E., Haberland, M., Reddy, T., Cournapeau, D., … SciPy 1.0 Contributors. (2020). SciPy 1.0: Fundamental algorithms for scientific computing in Python. Nature Methods, 17(3), 261–272. 10.1038/s41592-019-0686-2, 32015543 PMC7056644

[bib74] Wakana, S., Jiang, H., Nagae-Poetscher, L. M., van Zijl, P. C., & Mori, S. (2004). Fiber tract-based atlas of human white matter anatomy. Radiology, 230(1), 77–87. 10.1148/radiol.2301021640, 14645885

[bib75] Winkler, A. M., Ridgway, G. R., Douaud, G., Nichols, T. E., & Smith, S. M. (2016). Faster permutation inference in brain imaging. NeuroImage, 141, 502–516. 10.1016/j.neuroimage.2016.05.068, 27288322 PMC5035139

[bib76] Winkler, A. M., Ridgway, G. R., Webster, M. A., Smith, S. M., & Nichols, T. E. (2014). Permutation inference for the general linear model. NeuroImage, 92, 381–397. 10.1016/j.neuroimage.2014.01.060, 24530839 PMC4010955

[bib77] Woo, C. W., Krishnan, A., & Wager, T. D. (2014). Cluster-extent based thresholding in fMRI analyses: Pitfalls and recommendations. NeuroImage, 91, 412–419. 10.1016/j.neuroimage.2013.12.058, 24412399 PMC4214144

[bib78] Worsley, K. J., Evans, A. C., Marrett, S., & Neelin, P. (1992). A three-dimensional statistical analysis for CBF activation studies in human brain. Journal of Cerebral Blood Flow & Metabolism, 12(6), 900–918. 10.1038/jcbfm.1992.127, 1400644

[bib79] Worsley, K. J., Marrett, S., Neelin, P., & Evans, A. C. (1996). Searching scale space for activation in PET images. Human Brain Mapping, 4(1), 74–90. 10.1002/(SICI)1097-0193(1996)4:1<74::AID-HBM5>3.0.CO;2-M, 20408187

[bib80] Worsley, K. J., Marrett, S., Neelin, P., Vandal, A. C., Friston, K. J., & Evans, A. C. (1996). A unified statistical approach for determining significant signals in images of cerebral activation. Human Brain Mapping, 4(1), 58–73. 10.1002/(SICI)1097-0193(1996)4:1<58::AID-HBM4>3.0.CO;2-O, 20408186

[bib81] Yeo, B. T. T., Krienen, F. M., Sepulcre, J., Sabuncu, M. R., Lashkari, D., Hollinshead, M., … Buckner, R. L. (2011). The organization of the human cerebral cortex estimated by intrinsic functional connectivity. Journal of Neurophysiology, 106(3), 1125–1165. 10.1152/jn.00338.2011, 21653723 PMC3174820

[bib82] Yeung, A. W. K. (2018). An updated survey on statistical thresholding and sample size of fMRI studies. Frontiers in Human Neuroscience, 12, 1–7. 10.3389/fnhum.2018.00016, 29434545 PMC5790797

[bib83] Youden, W. J. (1950). Index for rating diagnostic tests. Cancer, 3(1), 32–35. 10.1002/1097-0142(1950)3:1<32::AID-CNCR2820030106>3.0.CO;2-3, 15405679

[bib84] Zhang, F., Wu, Y., Norton, I., Rigolo, L., Rathi, Y., Makris, N., & O’Donnell, L. J. (2018). An anatomically curated fiber clustering white matter atlas for consistent white matter tract parcellation across the lifespan. NeuroImage, 179, 429–447. 10.1016/j.neuroimage.2018.06.027, 29920375 PMC6080311

[bib85] Zhu, Q. (2020). On the performance of Matthews correlation coefficient (MCC) for imbalanced dataset. Pattern Recognition Letters, 136, 71–80. 10.1016/j.patrec.2020.03.030

