# Supplementary material for: Topological cluster statistic (TCS): Toward structural connectivity–guided fMRI cluster enhancement
Source: Netw Neurosci. 2024 Oct 1;8(3):902–25. doi: 10.1162/netn_a_00375 (PMC11424043; doi:10.1162/netn_a_00375)
Supplement: Supplementary file 1 [file netn-8-3-902-s001.pdf]

Supplementary Materials for

Topological Cluster Statistic (TCS): Towards structural-connectivity-guided fMRI cluster enhancement

S. Mansour L., C. Seguin, A. M. Winkler, S. Noble, and A. Zalesky

This PDF file includes:

Supplementary Text

Supplementary Figs. S1 to S16

Supplementary text

Replication of sensitivity evaluations for different sample sizes and CDTs

We repeated the evaluation of statistical power for alternative sample sizes. A log-linearly spaced set of sample size choices were selected for this evaluation ($N \in \{10, 20, 40, 80, 160, 320\}$, i.e. $N = \{10 \times 2^i | i \in \{0, 1, 2, 3, 4, 5\}\}$). Fig. S1 shows the true positive rate as a function of effect size for different sample sizes (replication of Fig. 3). As anticipated, a larger sample yielded improvements in sensitivity. Nevertheless, TCS provided improvement in statistical power for all sample sizes. The average improvement in sensitivity achieved by TCS compared to cluster-based statistics peaked at 2-5% for most tasks and sample sizes. In the specific case of the gambling task, with modest but distributed effect sizes, an improvement of up to 40% in mean sensitivity was observed.

Comparing improvements in TCS sensitivity for different sample sizes indicates that while TCS improved statistical power for all sample sizes, the extent of improvement varied by effect size and task. TCS improved detection of (i) relatively stronger effects ($0.5 < |d|$) in smaller sample sizes ($N = 10$ or $N = 20$), (ii) moderate effects ($0.4 < |d| < 0.6$) in medium sample sizes ($n = 40$ or $N = 80$), and (iii) weaker effects ($0.1 < |d| < 0.3$) in larger sample sizes ($N = 160$ or $N = 320$). These ranges mark effects that were harder to detect ($20\% < TPR < 80\%$) by cluster-based statistics and thus could have been improved by TCS. This suggests

that incorporating structural connectivity into the clustering process can enhance sensitivity, irrespective of sample size. Moreover, the results also indicate that the sensitivity improvements of TCS are rather minimal at very large sample sizes ($N > 300$) unless the effect under study is exceptionally weak (such as the gambling task).

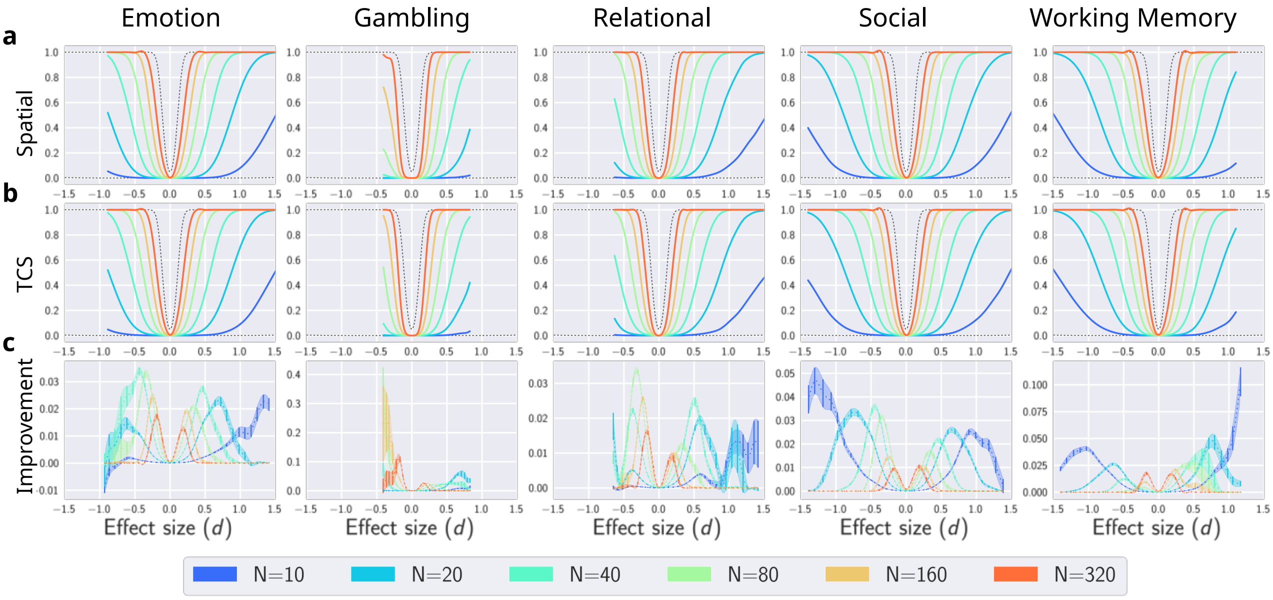

Fig. S1. Evaluation of sensitivity for various sample sizes. The statistical power (y-axis) against effect size (x-axis) is depicted for tests conducted with varying sample sizes ($N \in \{10, 20, 40, 80, 160, 320\}$). Average sensitivity is shown as a function of effect size in the putative ground truth. The first two rows (**a**, **b**) respectively depict the statistical power of the spatial cluster-based statistic and TCS. The lower dashed line depicts the estimated statistical power with Bonferroni correction in a sample of size $N = 10$. The higher dashed line depicts the statistical power without correction in a sample of size $N = 320$. The third row (**c**) depicts the differential gain in statistical power achieved by TCS over the cluster-based method. The shades depict 95% confidence intervals.

Similarly, these evaluations were repeated for a range of cluster-defining thresholds. Namely, evaluations were repeated for z-statistic thresholds of $z \in \{3.3, 2.8, 2.6, 2.0, 1.6\}$, which respectively correspond to $p \in \{0.001, 0.005, 0.01, 0.05, 0.1\}$. The results indicate that CDT can also influence the sensitivity of the inference. Generally, more lenient CDTs (smaller z) result in larger cluster sizes and higher sensitivity. However, lenient CDTs also create stronger nulls that make the hypothesis harder to reject. This can especially be seen in extremely lenient CDTs ($z \leq 2$) used in combination with spatial cluster inference. It is also evident that this increase in sensitivity can be at the cost of reduced specificity as the proportion of suprathreshold null effects ($d \approx 0$ in the ground truth) increase for more lenient CDTs. Nevertheless, the results

indicate that TCS can improve the sensitivity of inferences regardless of CDT. Furthermore, the results (considered alongside Figs. S5, S6, S9) suggest that for scenarios with generally weaker effects (as observed in the gambling task at lower sample sizes), TCS combined with a lenient CDT (e.g., $z = 2.0$) can provide optimal sensitivity enhancement while effectively controlling FPR.

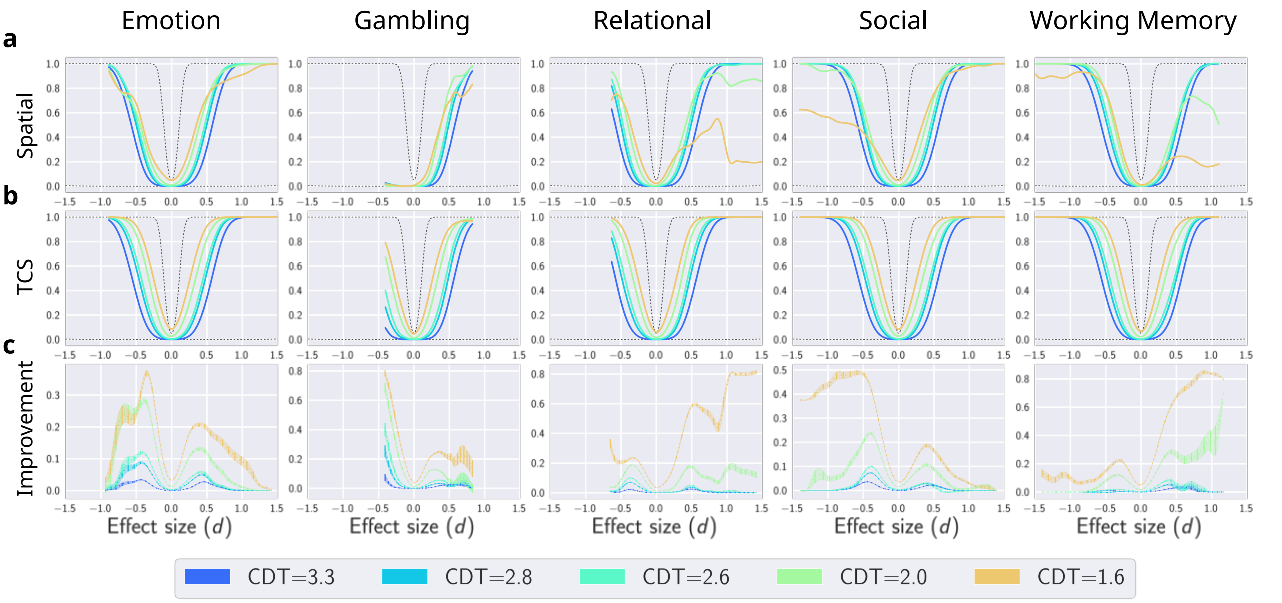

Fig. S2. Evaluation of sensitivity for various cluster-defining thresholds. The statistical power (y-axis) against effect size (x-axis) is depicted for tests conducted with varying CDTs ($z \in \{3.3, 2.8, 2.6, 2.0, 1.6\}$). Average sensitivity is shown as a function of effect size in the putative ground truth. The first two rows (**a**, **b**) respectively depict the statistical power of the spatial cluster-based statistic and TCS. The third row (**c**) depicts the differential gain in statistical power achieved by TCS over the cluster-based method. The shades depict 95% confidence intervals.

Replication of localized sensitivity gains for different sample sizes and CDTs

We additionally repeated assessments of localized sensitivity gains at every brainordinate for all tasks across different sample sizes ($N \in \{10, 20, 40, 80, 160, 320\}$). Figs. S3, S4 respectively show the local sensitivity projected on a glass brain, and a 2-dimensional distribution heatmap (replication of Fig. 4). These results demonstrate that large (>10%) local improvements are achieved across several sample sizes. A consistent pattern shows more local sensitivity improvements (warm colors in the glass brain projections) compared to fewer regions where TCS was less sensitive than cluster-based inference (cool colors). In larger samples, the local im-

provements shift to regions with smaller effect sizes (evident in Fig. S4). This reiterates that harder-to-detect effects benefit the most from TCS, regardless of sample size.

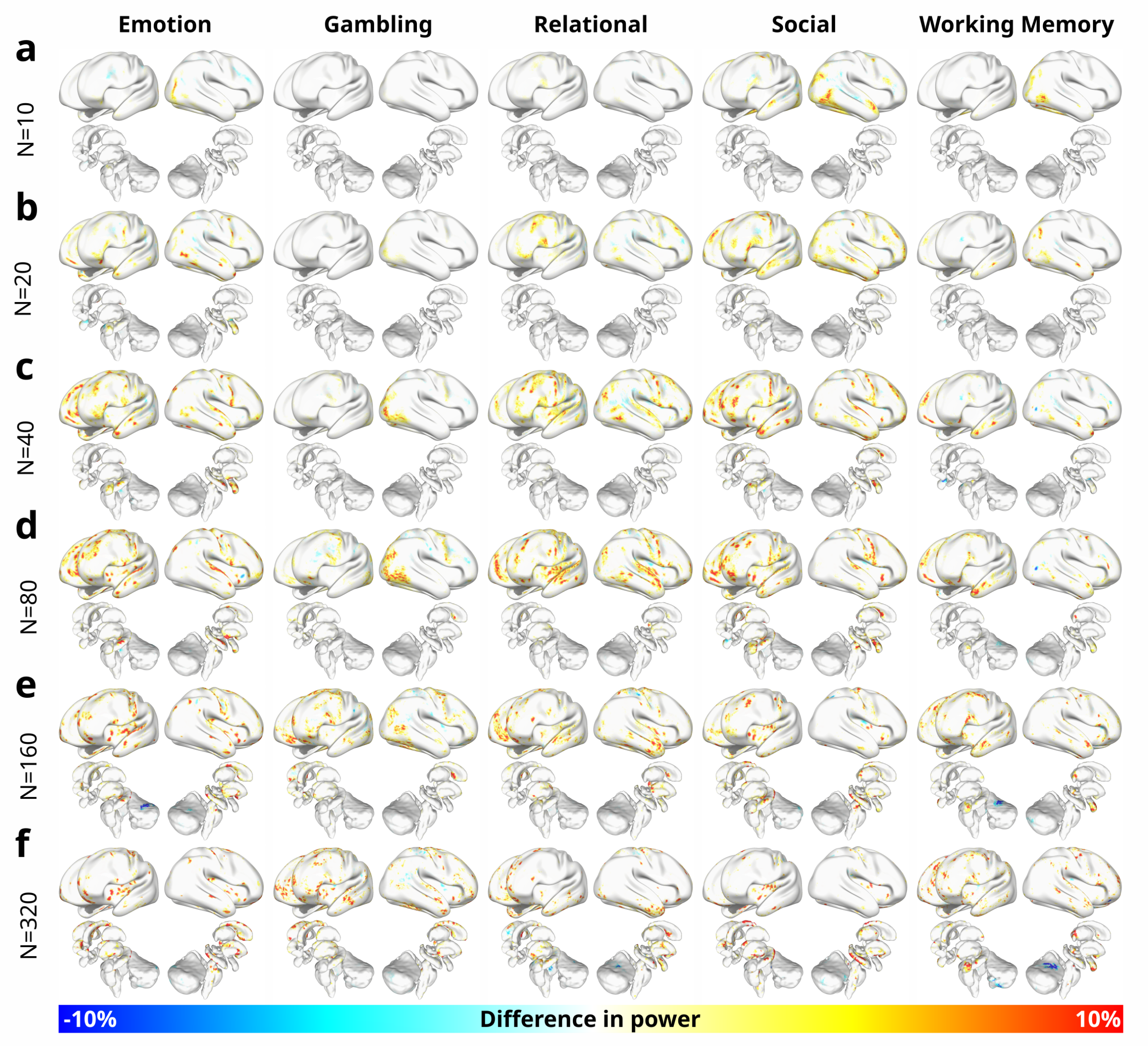

Fig. S3. Evaluation of localized sensitivity improvements for various sample sizes. The vertex-wise gains in sensitivity improvement of TCS compared to cluster-based statistics are projected onto a surface representation of the brain (similar to Fig. 4a). Rows (**a**, **b**, **c**, **d**, **e**, **f**) present the local gains for different sample sizes, respectively for $N \in \{10, 20, 40, 80, 160, 320\}$; whereas columns indicate different task contrasts. Warm colors indicate brainordinates where using TCS resulted in a sensitivity improvement. Conversely, cool colors indicate regions where cluster-based statistic was better powered. The color maps are capped at a 10% change in sensitivity.

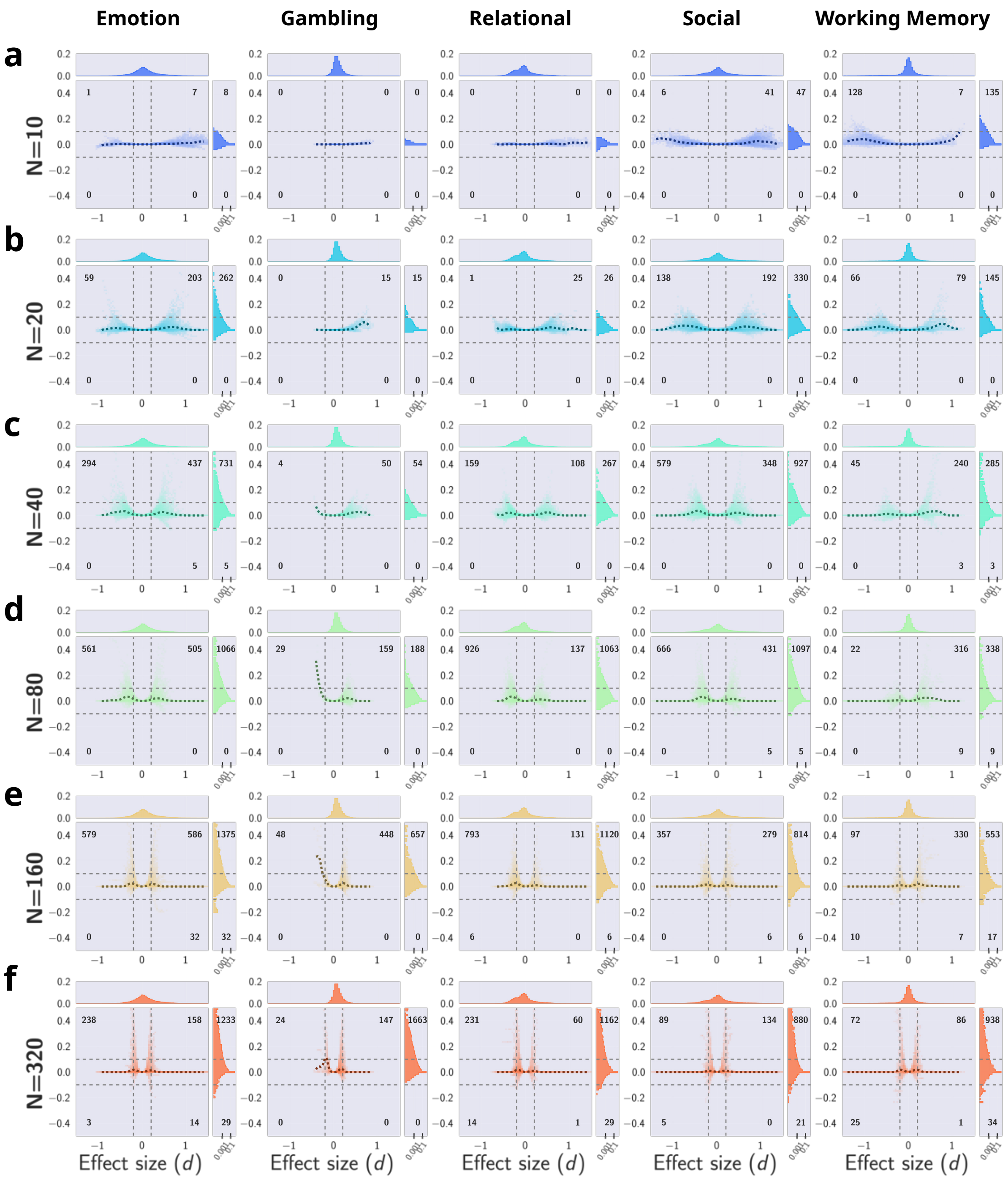

Fig. S4. Distribution of localized sensitivity improvements for various sample sizes. The vertex-wise gains in sensitivity improvement of TCS compared to cluster-based statistics are presented in the form of a 2D histogram (similar to Fig. 4b). Rows (**a**, **b**, **c**, **d**, **e**, **f**) present the distributions for different sample sizes, respectively for $N \in \{10, 20, 40, 80, 160, 320\}$; whereas columns indicate different task contrasts. The marginal distributions are depicted on the sides of the heatmap. Dashed vertical lines depict the small effect threshold ($|d| = 0.2$) and dashed horizontal lines depict a 10% change in sensitivity. The numbers of brainordinates exceeding both vertical and horizontal thresholds are presented on the four corners of the heatmap. The dashed curve depicts the mean improvements (similar to Fig. S1c). The marginal distribution of sensitivity improvement (histograms on the right side of the heatmap) is presented on a logarithmic scale.

We similarly repeated the localized sensitivity evaluations for different cluster-defining thresholds ($z \in \{3.3, 2.8, 2.6, 2.0, 1.6\}$) for all tasks. Figs. S5, S6 respectively show the local sensitivity projected on a glass brain, and a 2-dimensional distribution heatmap (replication of Fig. 4). These results further reiterate that TCS results in an increase in sensitivity regardless of the CDT. However, very lenient CDTs ($z \leq 2$) should nevertheless be avoided as they lead to a severe increase in false positive rates (localized improvement at very small effects where $d \approx 0$).

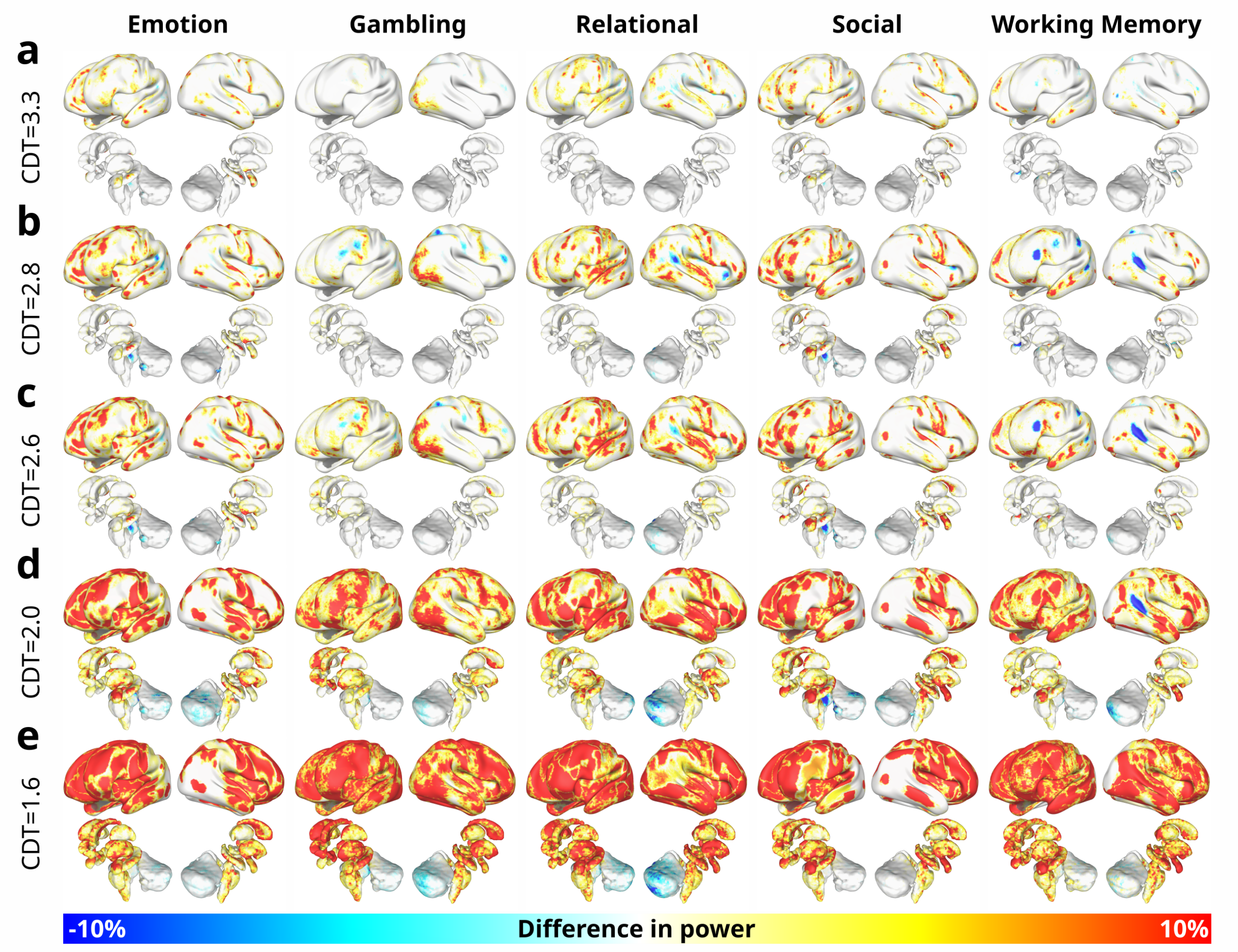

Fig. S5. Evaluation of localized sensitivity improvements for various cluster-defining thresholds. The vertex-wise gains in sensitivity improvement of TCS compared to cluster-based statistics are projected onto a surface representation of the brain (similar to Fig. 4a). Rows (**a**, **b**, **c**, **d**, **e**) present the local gains for different CDTs, respectively for $z \in \{3.3, 2.8, 2.6, 2.0, 1.6\}$; whereas columns indicate different task contrasts. Warm colors indicate brainordinates where using TCS resulted in a sensitivity improvement. Conversely, cool colors indicate regions where cluster-based statistic was better powered. The color maps are capped at a 10% change in sensitivity.

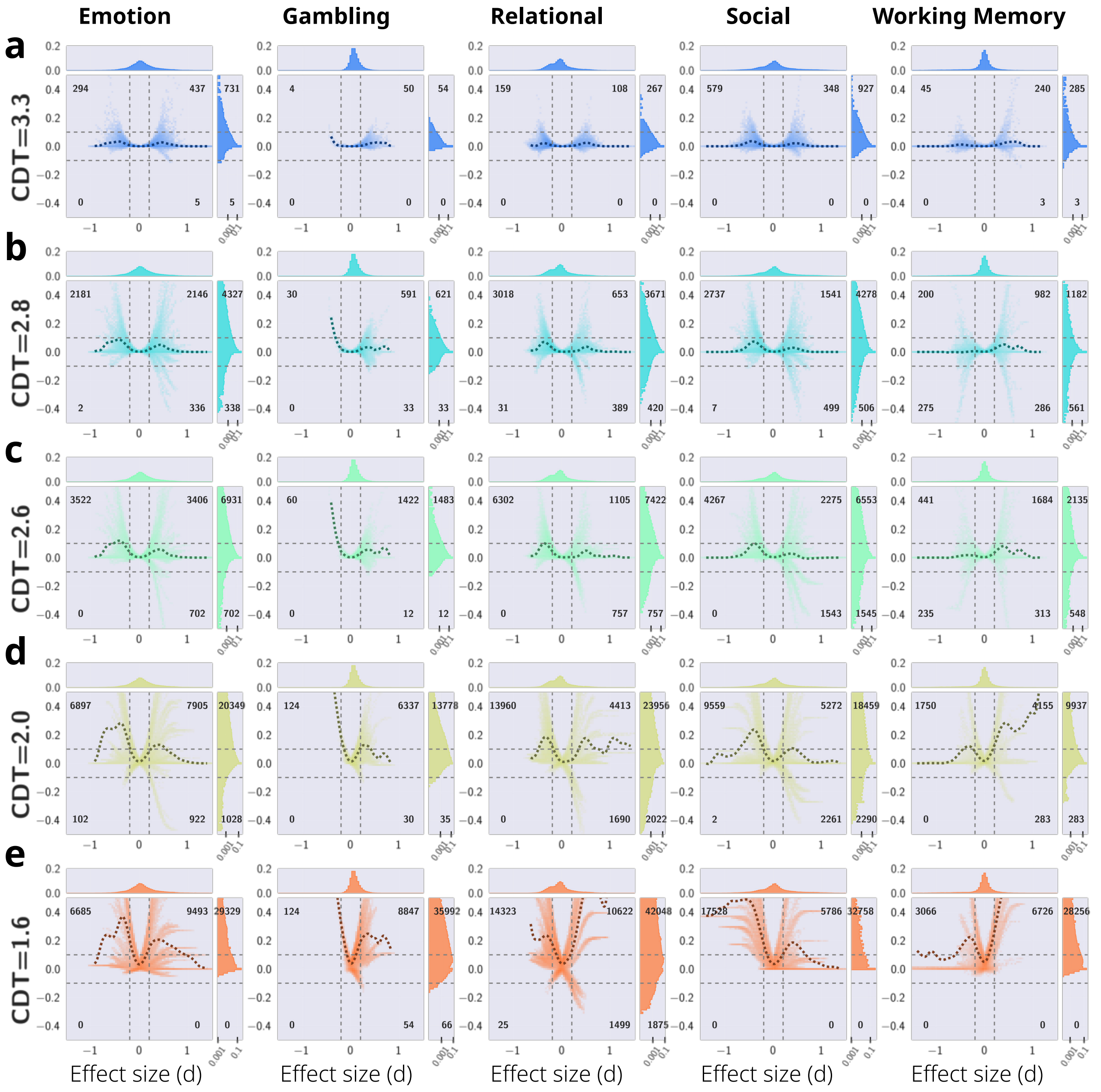

Fig. S6. Distribution of localized sensitivity improvements for various cluster-defining thresholds. The vertex-wise gains in sensitivity improvement of TCS compared to cluster-based statistics are presented in the form of a 2D histogram (similar to Fig. 4b). Rows (**a**, **b**, **c**, **d**, **e**) present the distributions for different CDTs, respectively for $z \in \{3.3, 2.8, 2.6, 2.0, 1.6\}$; whereas columns indicate different task contrasts. The marginal distributions are depicted on the sides of the heatmap. Dashed vertical lines depict the small effect threshold ($|d| = 0.2$) and dashed horizontal lines depict a 10% change in sensitivity. The numbers of brainordinates exceeding both vertical and horizontal thresholds are presented on the four corners of the heatmap. The dashed curve depicts the mean improvements (similar to Fig. S1c). The marginal distribution of sensitivity improvement (histograms on the right side of the heatmap) is presented on a logarithmic scale.

Replication of sensitivity vs. specificity evaluations for different sample sizes and CDTs

We repeated assessments of the classifier informedness for a range of alternative sample sizes ($N \in \{10, 20, 40, 80, 160, 320\}$) and CDTs ($z \in \{3.3, 2.8, 2.6, 2.0, 1.6\}$). Figs. S7, S8 reproduce the results presented in Fig. 5 for different sample sizes and CDTs respectively. Importantly, these supplementary evaluations highlight that the improvements in sensitivity achieved by TCS are consistently resulting in a more informed classifier. Essentially, in all cases, the informedness improvements presented in Fig. S7c remain strictly positive. As such, the improvements in sensitivity were not at the cost of worse reductions in specificity, regardless of the study sample size or cluster-defining threshold.

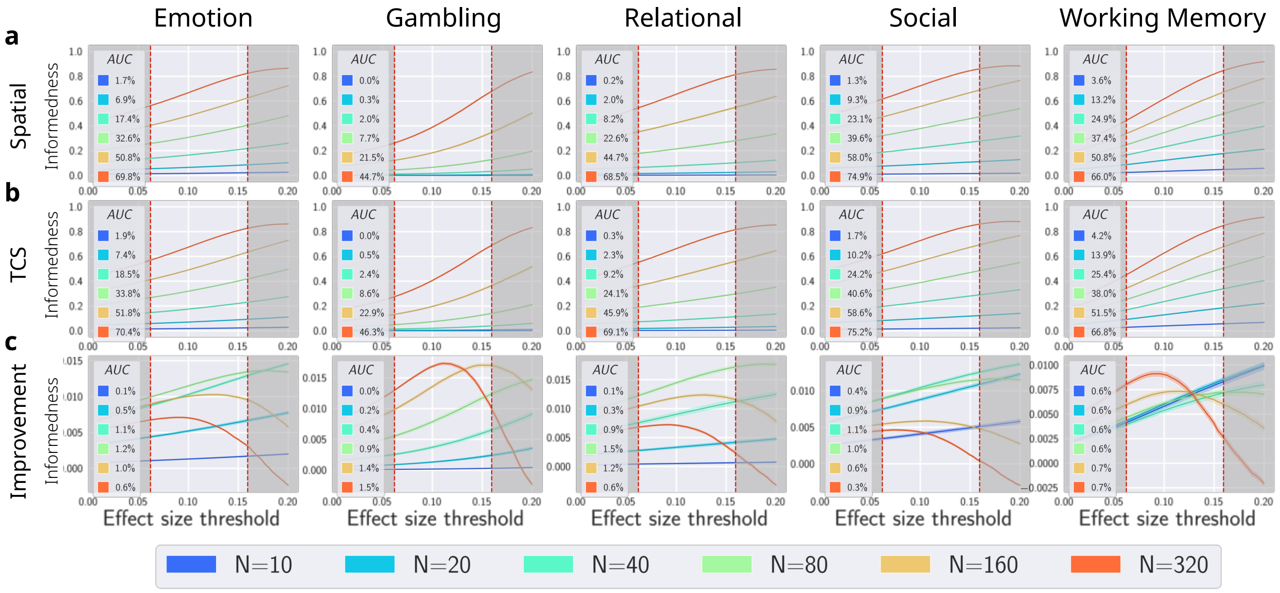

Fig. S7. Informedness of clustering inferences at different sample sizes. Significant clusters surviving correction were treated as labels for binary classification (significant vs. null). The bookmaker informedness index was utilized to compare performance. Repetitions with different sample sizes are color-coded. The first two rows (**a**,**b**) respectively depict informedness of spatial clustering and TCS as a function of the binarization threshold. Informedness was summarized by the normalized area under the curve (AUC) that averages informedness across thresholds, thus providing a threshold-independent comparison. The third row (**c**) indicates the informedness improvement yielded by TCS. The shaded lines indicate the 95% confidence interval. The dashed vertical lines indicate the required effect for statistical significance ($\alpha = 5\%$) in the putative ground truth sample (N=1000) with (right) and without (left) a Bonferroni correction. The right line indicates a very stringent binarization threshold, and the left line gives a more lenient threshold. AUC was computed for values falling between the two dashed lines.

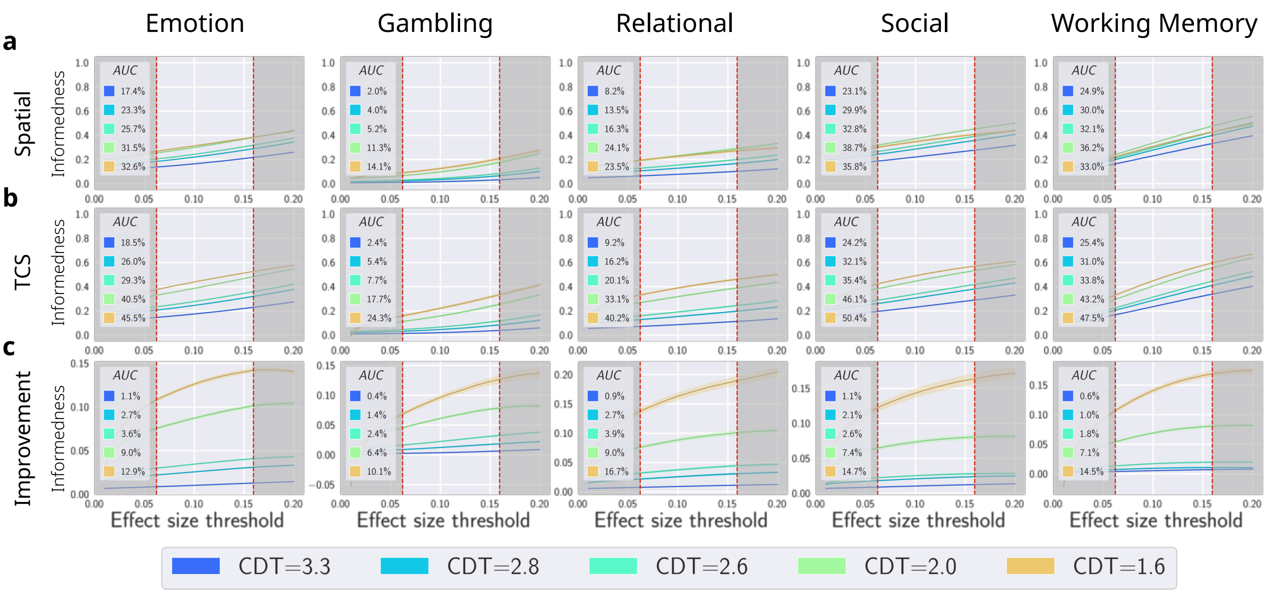

Fig. S8. Informedness of clustering inferences at different cluster-defining thresholds. Significant clusters surviving correction were treated as labels for binary classification (significant vs. null). The bookmaker informedness index was utilized to compare performance. Repetitions with different sample sizes are color-coded. The first two rows (**a**,**b**) respectively depict informedness of spatial clustering and TCS as a function of the binarization threshold. Informedness was summarized by the normalized area under the curve (AUC) that averages informedness across thresholds, thus providing a threshold-independent comparison. The third row (**c**) indicates the informedness improvement yielded by TCS. The shaded lines indicate the 95% confidence interval. The dashed vertical lines indicate the required effect for statistical significance ($\alpha = 5\%$) in the putative ground truth sample (N=1000) with (right) and without (left) a Bonferroni correction. The right line indicates a very stringent binarization threshold, and the left line gives a more lenient threshold. AUC was computed for values falling between the two dashed lines.

Other evaluations of inference performance

Here, we provide other evaluations of inference performance to further compare TCS with cluster-based inference methods. To this end, another metric was computed to quantify the performance of both inference approaches. Namely, the true positive rate and false positive rate were computed for a range of CDTs ($z \in \{3.3, 2.8, 2.6, 2.0, 1.6\}$) and sample sizes ($N \in \{10, 20, 40, 80, 160, 320\}$). For each task and sample size, a line plot of TPR against FPR at different CDTs resulted in a receiver operating characteristic (ROC) curve. The normalized partial area under the curve (AUC) was used to quantify inference performance. An AUC closer to one (100%) indicates a higher performance. Fig. S9 shows the ROC curves across the parameter space. AUC is presented as labels for each plot. The results indicate that TCS

consistently results in higher AUCs for all combinations of sample size and task. This further recapitulates that TCS improves the inference performance compared to cluster-based statistics. Moreover, these results approve previous suggestions that more stringent CDTs should be used with larger samples to control false positive rates (74).

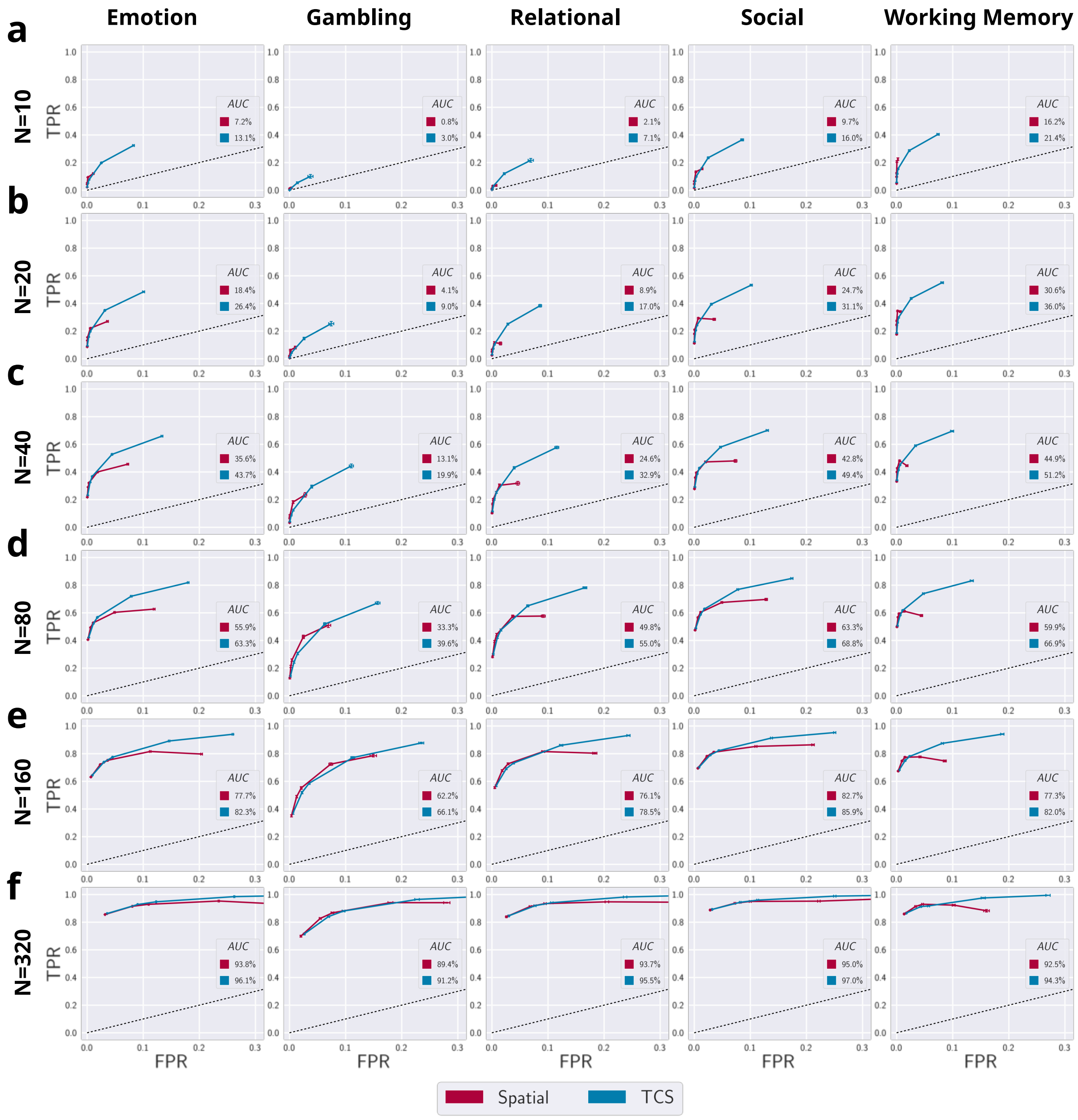

Fig. S9. Evaluating inference performance by the area under ROC curve. Significant clusters surviving correction were treated as labels for binary classification (significant vs. null). Line plots indicate the ROC curve (change in true positive rate against false positive rate) for TCS (teal) and spatial cluster statistic (violet). Rows separate the evaluations at different sample sizes. Columns separate different tasks. Legends indicate the area under ROC curve.

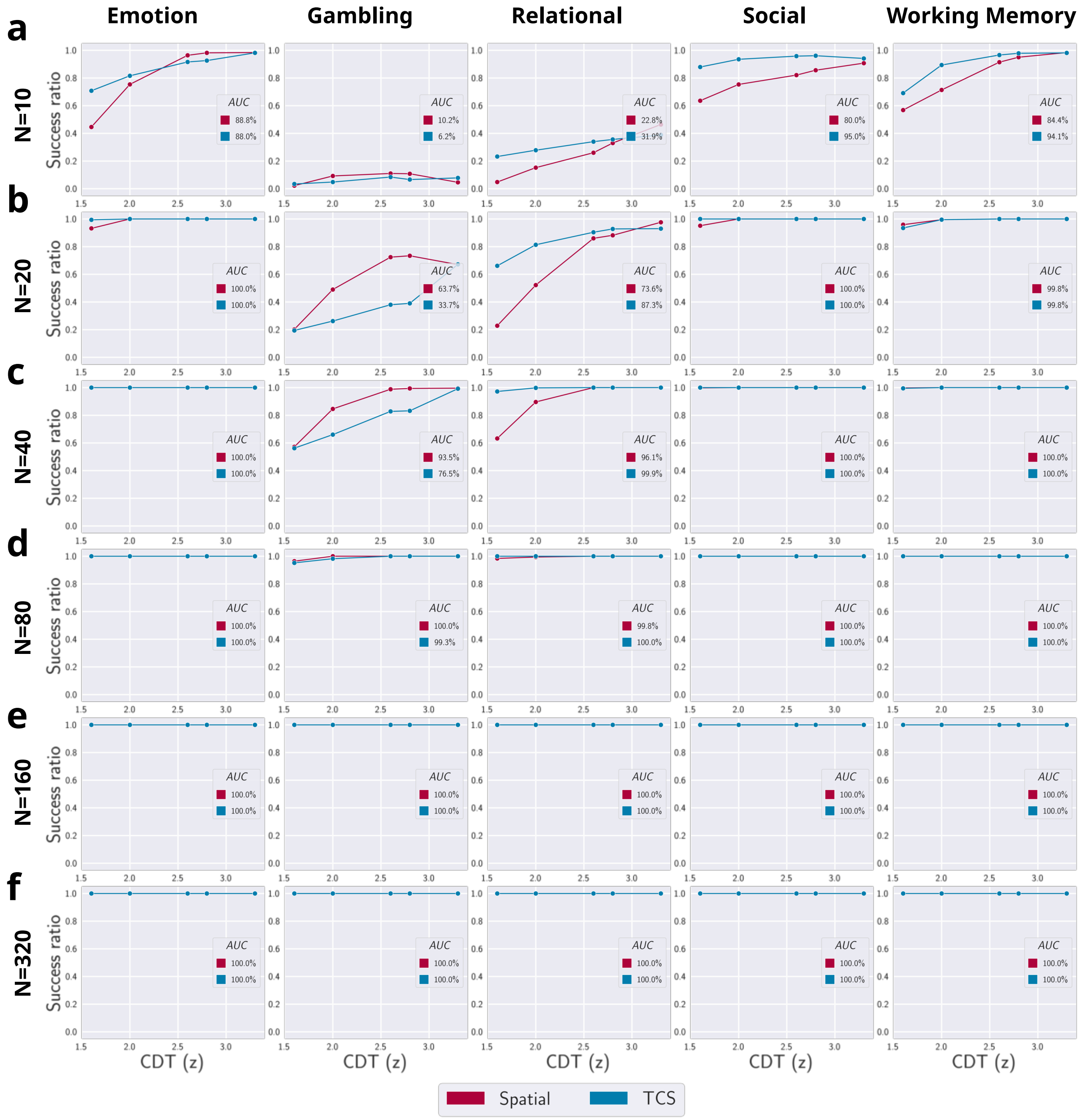

Fig. S10. Evaluating inference success ratio. The likelihood of detecting effects was quantified based on the ratio of all repetitions that found a significant effect for TCS (teal) and spatial cluster statistic (violet). Columns separate results by task contrasts. Rows separate the results at different sample sizes ($N \in \{10, 20, 40, 80, 160, 320\}$). The x-axes separate repetitions at different CDTs ($z \in \{3.3, 2.8, 2.6, 2.0, 1.6\}$). In addition to success ratios (presented as scattered points), the area under the connecting curve is provided that summarizes the difference for each pair of sample size and task contrast.

Finally, in another set of evaluations, we quantified the number of times (over 500 repetitions) that each inference approach was able to detect any cluster of significant activation (FWER corrected at the level of clusters). Division of this number by the total repetitions (500) provides a measure of the detection success ratio. The success ratio quantifies the like-

lihood of detecting any effects with either TCS or spatial cluster-based approach at any given choice of cluster-defining threshold and sample size. Fig. S10 presents a comparison of success ratios for all tasks on a range of CDTs ($z \in \{3.3, 2.8, 2.6, 2.0, 1.6\}$) and sample sizes ($N \in \{10, 20, 40, 80, 160, 320\}$). Generally, the findings indicate that for a conventional CDT ($z = 3.3$) or large enough samples ($N \geq 80$) there is little to no difference between the success ratios. However, for more lenient CDTs in smaller sample sizes differences were observable which favored TCS in the Relational, Social, and Working Memory tasks, but favored spatial clustering in the Gambling task. This may be an indication that the activation patterns in the Gambling task may be less distributed over spatially disjoint but anatomically connected regions.

4.15 Detailed comparison and anatomical networks for other tasks

In the main results, a detailed comparison of TCS and the conventional cluster-based inference was provided for a single repetition of the emotion task (presented in Fig. 6). This comparison also included atlas connectograms and anatomical network visualizations generated for TCS. For the sake of completeness, similar comparisons for the other four remaining tasks are provided in Figs. S11, S12, S13, S14. Furthermore, given the observed similarities in identifying large spatial activations, Fig. S15 provides an additional color-coded visualization for the five task contrasts in Figs. 6, S11, S12, S13, S14 that illustrates the spatial overlap between the two methods and highlights regions detected exclusively by each method. These results reiterate the benefits of TCS in (i) capturing smaller spatial effects, (ii) indicating that many fragmented spatial effects are in fact part of a larger group of anatomically linked clusters, and (iii) presenting interpretable networks that can facilitate locating relevant anatomical pathways implicated in a particular task.

In the concrete, TCS helped unravel the diversity of effects across HCP tasks. The social task showed the most widespread activations that were supported by a vast underlying network of anatomical connections. In contrast, the gambling task showed relatively sparse activations and supporting connectivity (Fig.S16 shows how a lenient CDT can better detect smaller effects of this particular task). The relational task featured the most connected negative activations, whereas the emotion task features the most connected positive activations. Finally, the working memory task was unique in that it mostly featured negative activations across the connected oc-

cipital lobe, which, in turn, shared connectivity with positive activations occurring elsewhere in the cortex. Furthermore, across different tasks and brain regions, many homotopic connections (linking mirroring brain regions from left and right hemispheres) are evidently linking inter-cortical effects. This verifies that observed symmetries in brain activation maps are supported by the underlying connections through commissural fibers. This summary of task-based effects in the HCP illustrates how TCS enables the interpretation of not only the spatial locations of effects but also the underlying scaffolding that supports these activations.

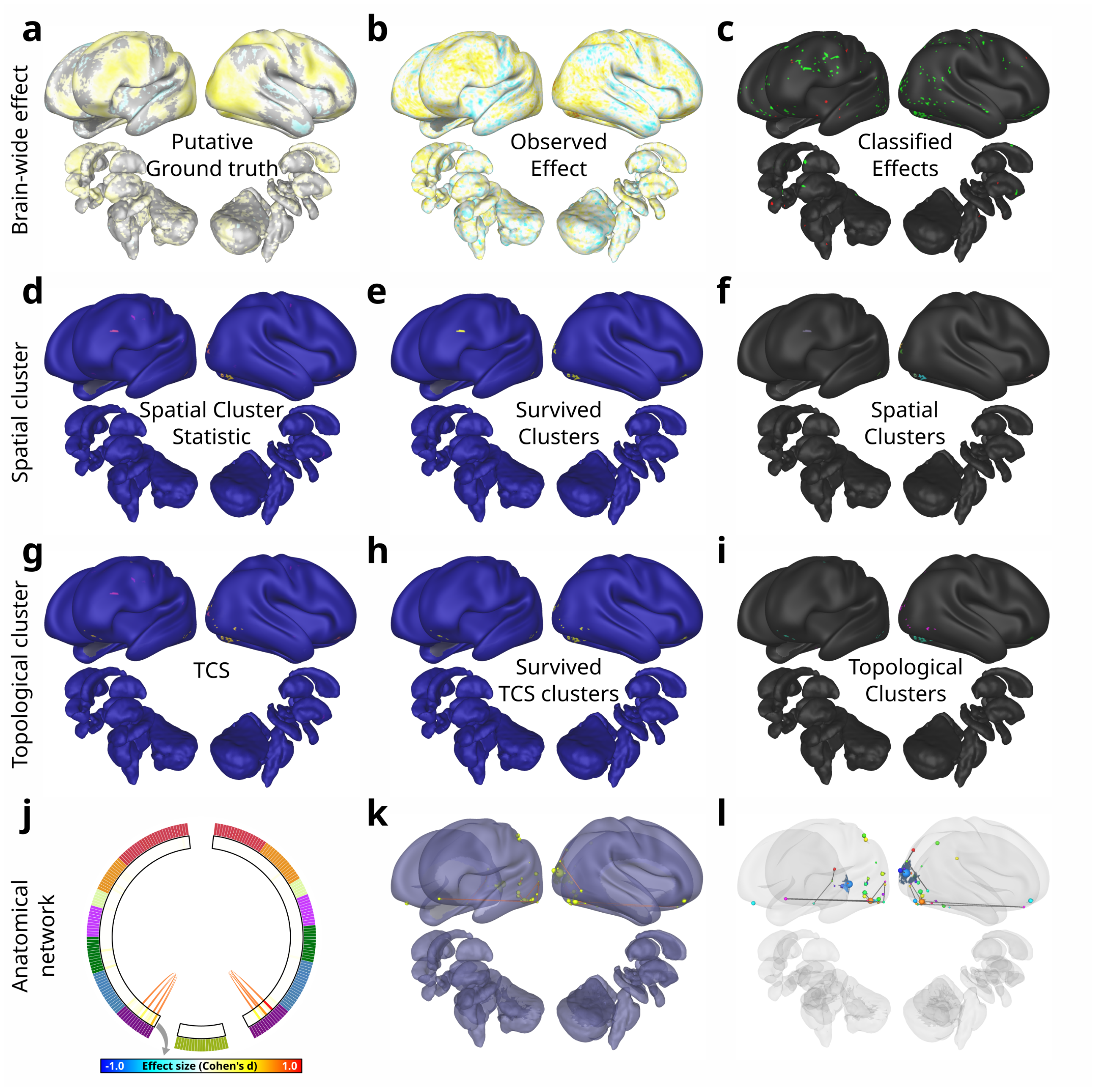

Fig. S11. Illustrating benefits of TCS on the gambling task. **a** The putative ground truth effect size ($N = 994$) for the gambling task. The effects not reaching significance ($p \geq 0.05$) are grayed out. **b** The effects from the same task, observed over a smaller sample ($N = 40$). **c** The effects are colored to distinguish between detectable true positives (green), false positives (red), and regions where the observed effect falls below the cluster-forming threshold (black). **d** The spatial cluster-based statistic value for all suprathreshold spatial clusters. **e** The spatial clusters that survive non-parametric FWER correction at the level of clusters. **f** Clusters colored by spatial contiguity. **g** TCS for suprathreshold regions. **h** TCS clusters that survive correction. **i** Clusters colored by topological contiguity. **j** A chord diagram of anatomical connectivity between TCS clusters downsampled to a brain atlas. The diagram is broken into the subcortex and cerebellum (bottom), and the left and right cortices (colored by their respective 7 RSN networks in the outer layer). The inner layer depicts the mean effect size. **k** Anatomical connectivity between spatially disjoint effect clusters. **l** The spanning tree covering the key anatomical connections based on maximal pairwise effect magnitude.

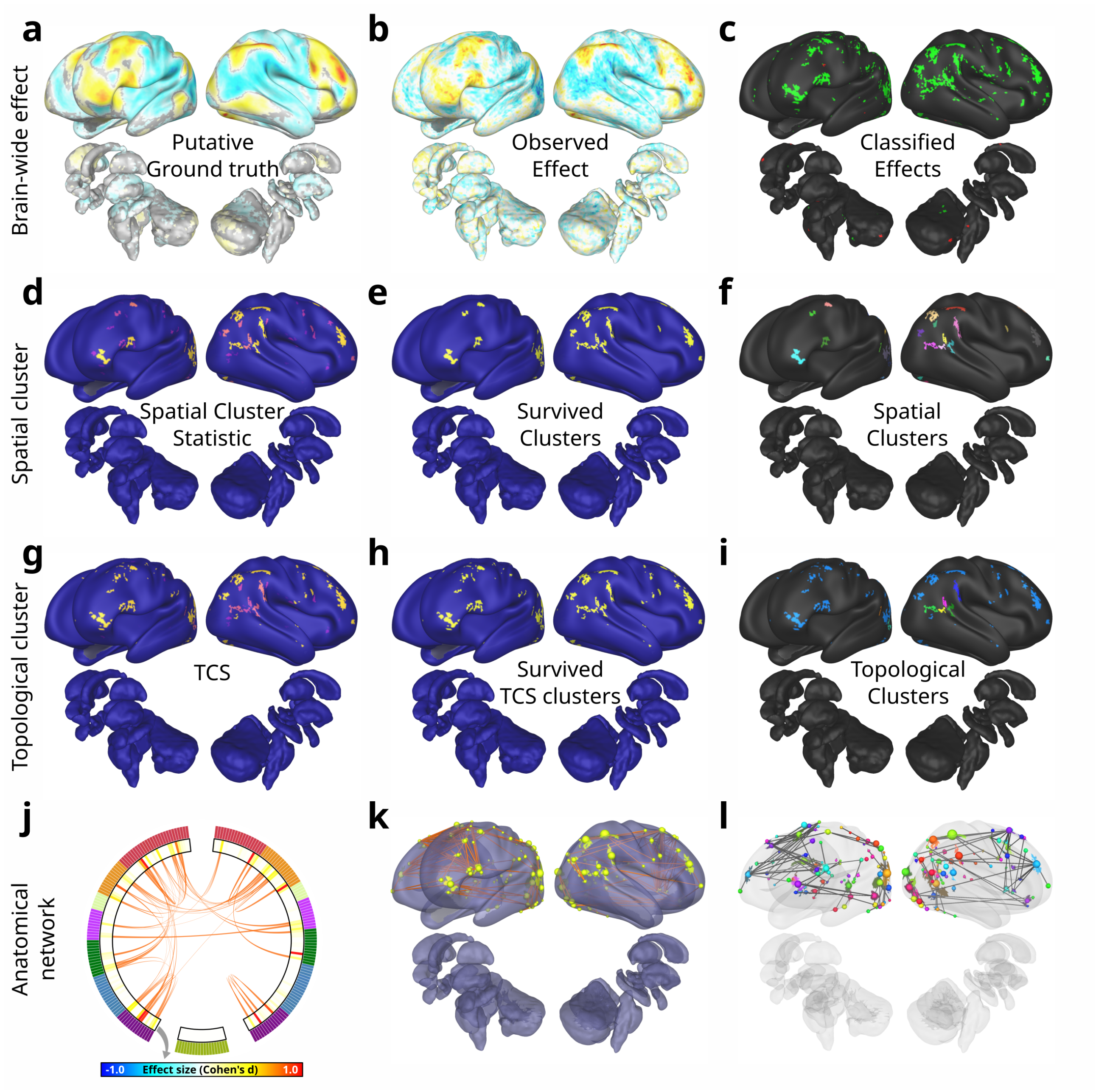

Fig. S12. Illustrating benefits of TCS on the relational task. **a** The putative ground truth effect size ($N = 983$) for the relational task. The effects not reaching significance ($p \geq 0.05$) are grayed out. **b** The effects from the same task, observed over a smaller sample ($N = 40$). **c** The effects are colored to distinguish between detectable true positives (green), false positives (red), and regions where the observed effect falls below the cluster-forming threshold (black). **d** The spatial cluster-based statistic value for all suprathreshold spatial clusters. **e** The spatial clusters that survive non-parametric FWER correction at the level of clusters. **f** Clusters colored by spatial contiguity. **g** TCS for suprathreshold regions. **h** TCS clusters that survive correction. **i** Clusters colored by topological contiguity. **j** A chord diagram of anatomical connectivity between TCS clusters downsampled to a brain atlas. The diagram is broken into the subcortex and cerebellum (bottom), and the left and right cortices (colored by their respective 7 RSN networks in the outer layer). The inner layer depicts the mean effect size. **k** Anatomical connectivity between spatially disjoint effect clusters. **l** The spanning tree covering the key anatomical connections based on maximal pairwise effect magnitude.

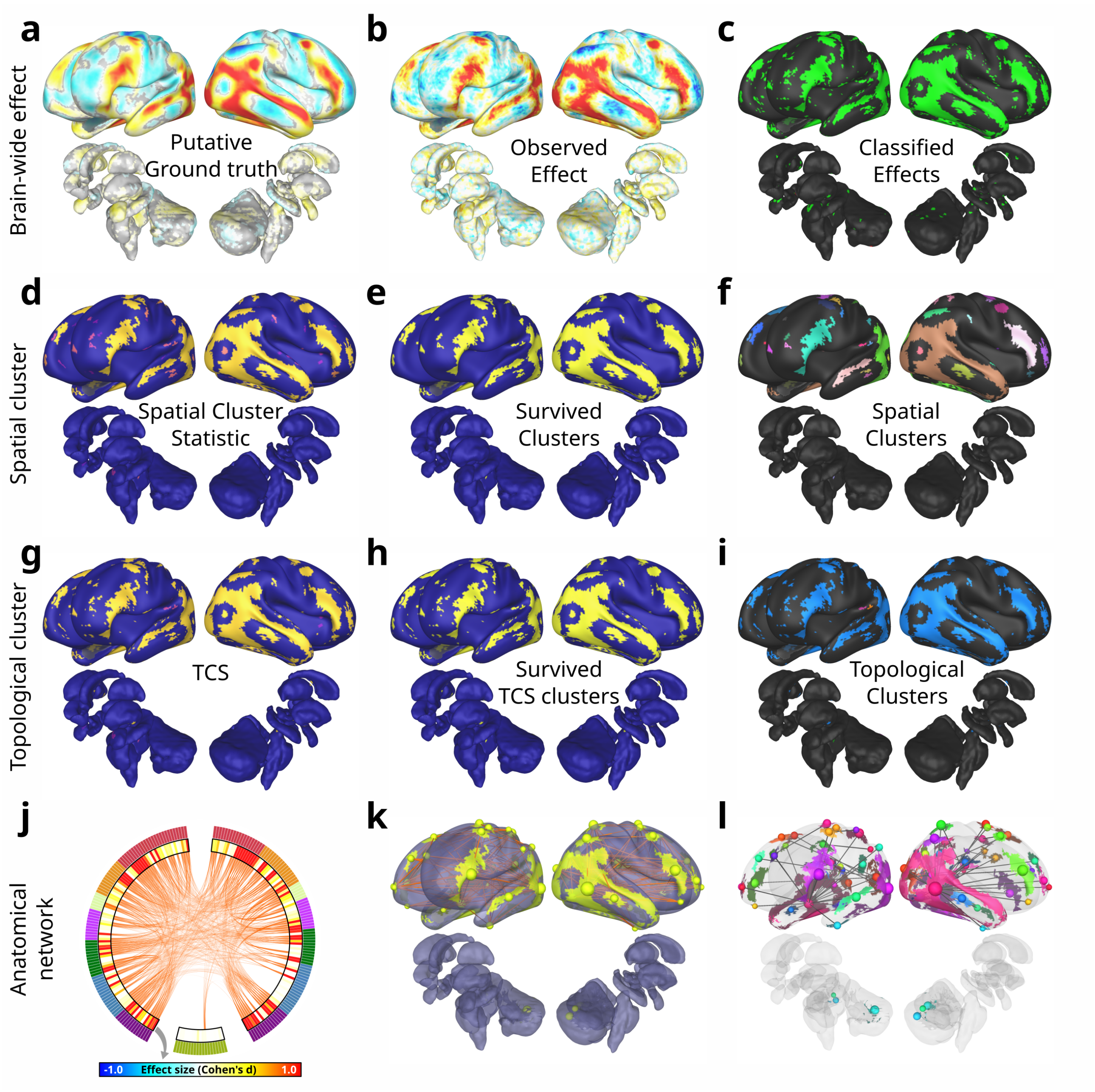

Fig. S13. Illustrating benefits of TCS on the social task. **a** The putative ground truth effect size ($N = 991$) for the social task. The effects not reaching significance ($p \geq 0.05$) are grayed out. **b** The effects from the same task, observed over a smaller sample ($N = 40$). **c** The effects are colored to distinguish between detectable true positives (green), false positives (red), and regions where the observed effect falls below the cluster-forming threshold (black). **d** The spatial cluster-based statistic value for all suprathreshold spatial clusters. **e** The spatial clusters that survive non-parametric FWER correction at the level of clusters. **f** Clusters colored by spatial contiguity. **g** TCS for suprathreshold regions. **h** TCS clusters that survive correction. **i** Clusters colored by topological contiguity. **j** A chord diagram of anatomical connectivity between TCS clusters downsampled to a brain atlas. The diagram is broken into the subcortex and cerebellum (bottom), and the left and right cortices (colored by their respective 7 RSN networks in the outer layer). The inner layer depicts the mean effect size. **k** Anatomical connectivity between spatially disjoint effect clusters. **l** The spanning tree covering the key anatomical connections based on maximal pairwise effect magnitude.

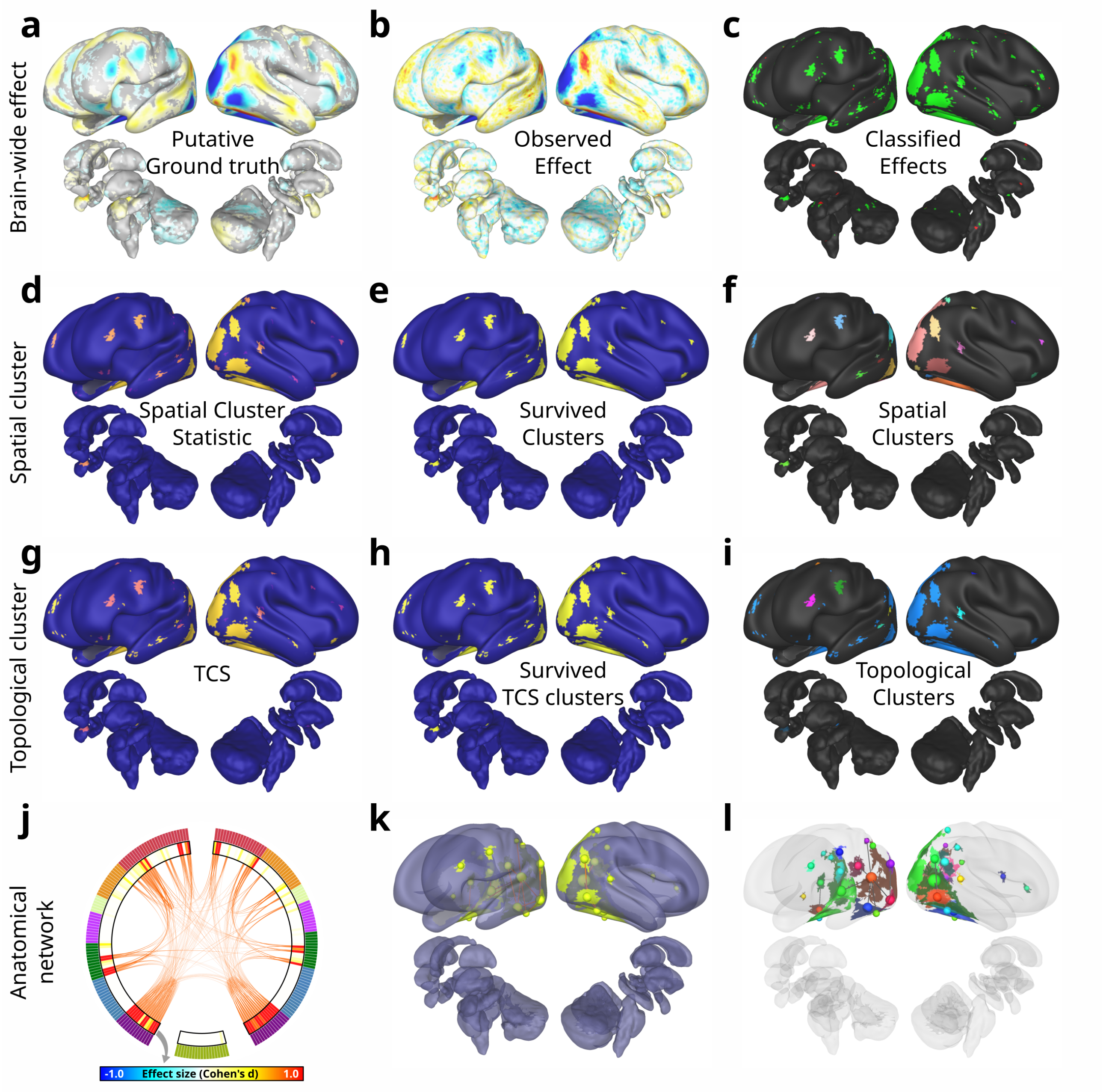

Fig. S14. Illustrating benefits of TCS on the working memory task. **a** The putative ground truth effect size ($N = 992$) for the working memory task. The effects not reaching significance ($p \geq 0.05$) are grayed out. **b** The effects from the same task, observed over a smaller sample ($N = 40$). **c** The effects are colored to distinguish between detectable true positives (green), false positives (red), and regions where the observed effect falls below the cluster-forming threshold (black). **d** The spatial cluster-based statistic value for all suprathreshold spatial clusters. **e** The spatial clusters that survive non-parametric FWER correction at the level of clusters. **f** Clusters colored by spatial contiguity. **g** TCS for suprathreshold regions. **h** TCS clusters that survive correction. **i** Clusters colored by topological contiguity. **j** A chord diagram of anatomical connectivity between TCS clusters downsampled to a brain atlas. The diagram is broken into the subcortex and cerebellum (bottom), and the left and right cortices (colored by their respective 7 RSN networks in the outer layer). The inner layer depicts the mean effect size. **k** Anatomical connectivity between spatially disjoint effect clusters. **l** The spanning tree covering the key anatomical connections based on maximal pairwise effect magnitude.

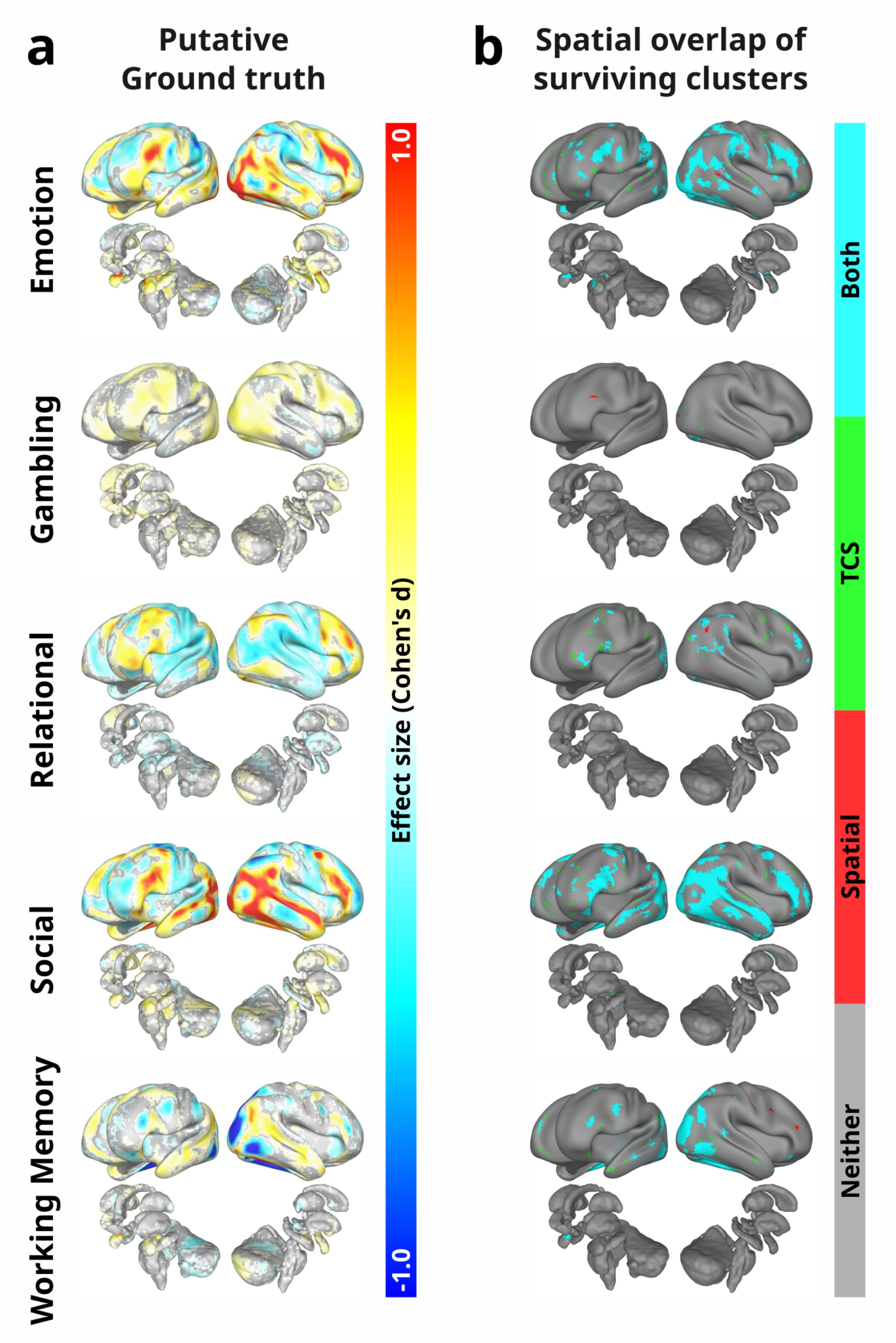

Fig. S15. The overlap of effects captured by TCS and spatial cluster-based statistics. **a** The putative ground truth effect size for the same five tasks (respectively presented on Figs. 6, S11, S12, S13, S14). The effects not reaching significance ($p \geq 0.05$) are grayed out. **b** Spatial overlap of areas detected by either approach. In brief, task effects are inferred in a smaller sample ($N = 40$); cluster correction is performed using either spatial cluster-based statistics or TCS; the clusters that survive non-parametric FWER correction at the level of clusters are recorded. A color-coded surface projection is used to distinguish between overlapping clusters that survive correction by both methods (cyan), as well as those exclusively detected by either the spatial statistic (red) or TCS (green).

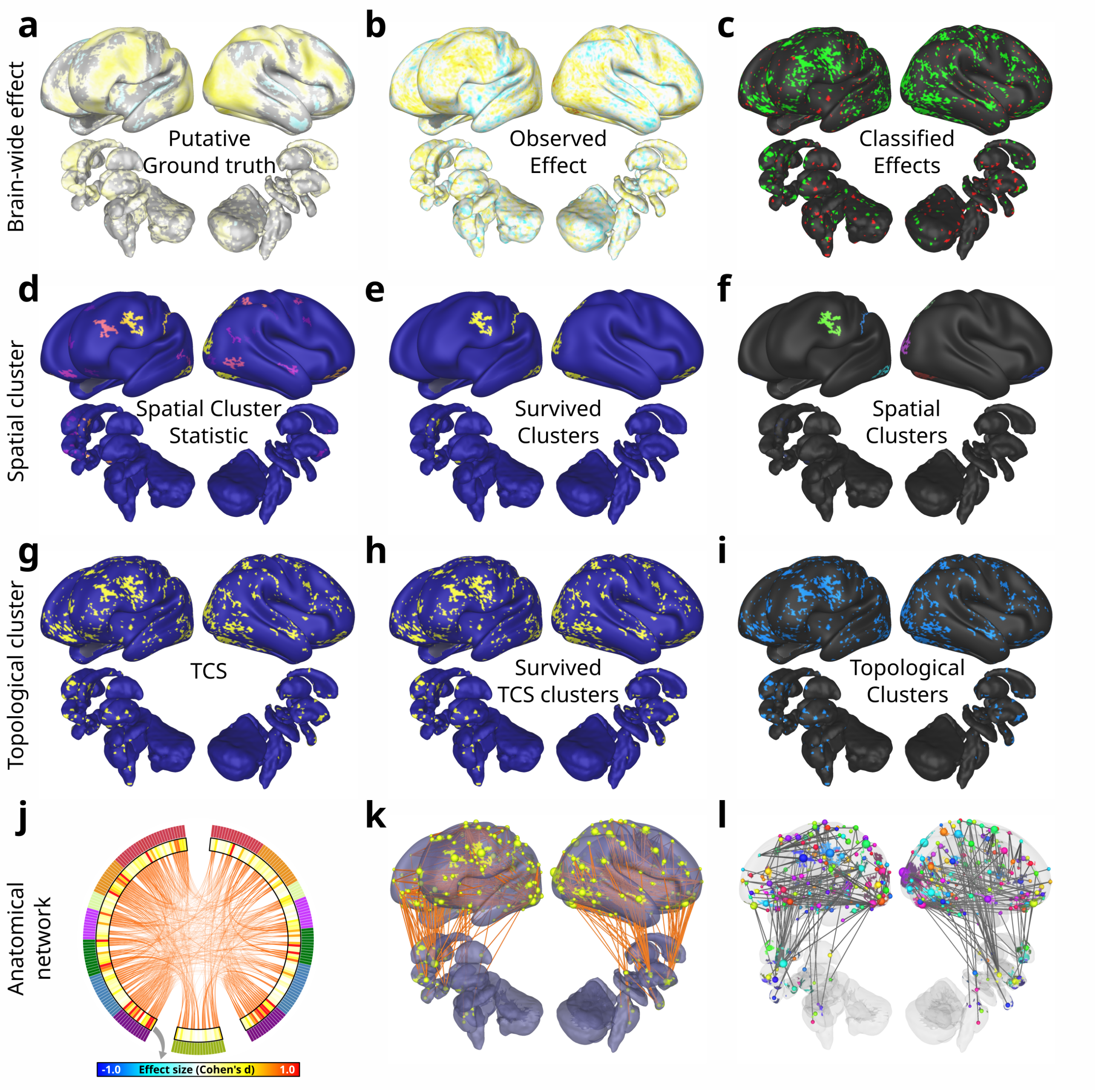

Fig. S16. Illustrating benefits of TCS on the gambling task with a lenient cluster defining threshold of $z = 2.0$. **a** The putative ground truth effect size ($N = 994$) for the gambling task. The effects not reaching significance ($p \geq 0.05$) are grayed out. **b** The effects from the same task, observed over a smaller sample ($N = 40$). **c** The effects are colored to distinguish between detectable true positives (green), false positives (red), and regions where the observed effect falls below the cluster-forming threshold ($z = 2.0$) (black). **d** The spatial cluster-based statistic value for all suprathreshold spatial clusters. **e** The spatial clusters that survive non-parametric FWER correction at the level of clusters. **f** Clusters colored by spatial contiguity. **g** TCS for suprathreshold regions. **h** TCS clusters that survive correction. **i** Clusters colored by topological contiguity. **j** A chord diagram of anatomical connectivity between TCS clusters downsampled to a brain atlas. The diagram is broken into the subcortex and cerebellum (bottom), and the left and right cortices (colored by their respective 7 RSN networks in the outer layer). The inner layer depicts the mean effect size. **k** Anatomical connectivity between spatially disjoint effect clusters. **l** The spanning tree covering the key anatomical connections based on maximal pairwise effect magnitude.
